# Antimicrobial resistance in nontyphoidal *Salmonella* associated with multistate outbreaks linked to backyard poultry, United States, 2018–2023

**DOI:** 10.3389/fpubh.2026.1854943

**Published:** 2026-06-12

**Authors:** Marisa Wong, Laura Ford, G. Sean Stapleton, Meseret G. Birhane, Louise K. Francois Watkins, Zachary Ellison, Justin Kim, Jason P. Folster, Hayat Caidi, Megin Nichols, Cary Krug, Allison E. James

**Affiliations:** 1 Division of Foodborne, Waterborne, and Environmental Diseases, Centers for Disease Control and Prevention, Atlanta, GA, United States; 2 Oak Ridge Institute for Science and Education, Oak Ridge, TN, United States; 3 ASRT, Inc., Smyrna, GA, United States

**Keywords:** antimicrobial resistance, backyard poultry, One Health, *Salmonella*, zoonotic disease

## Abstract

**Introduction:**

State and local health departments and the Centers for Disease Control and Prevention (CDC) routinely investigate multistate outbreaks of salmonellosis associated with backyard poultry (BYP). Here, we describe antimicrobial resistance in multistate outbreaks of nontyphoidal salmonellosis associated with BYP from 2018 to 2023.

**Methods:**

We analyzed patient and outbreak data from CDC’s National Antimicrobial Resistance Monitoring System and System for Enteric Disease Response, Investigation, and Coordination databases. Isolate resistance was determined by antimicrobial susceptibility testing or predicted by whole genome sequencing. We classified isolates as resistant, multi-drug resistant (MDR; resistant to ≥3 antimicrobial classes), or having clinically relevant resistance (CRR; resistant to ampicillin, azithromycin, ceftriaxone, or trimethoprim-sulfamethoxazole; or nonsusceptibility to ciprofloxacin), and outbreaks as resistant, MDR, or CRR if ≥3 isolates and ≥10% of isolates met the corresponding resistance definitions. Statistical analyses comparing patient variables, outbreak size, and annual number of outbreaks with resistance status were performed.

**Results:**

Among 78 multistate BYP-associated salmonellosis outbreaks, 36 (46%) exhibited resistance, 10 (13%) were classified as MDR, and 12 (15%) were classified as CRR. Enteritidis was the most frequent serotype, causing 22 (28%) outbreaks; three were resistant (two CRR only outbreaks, one CRR and MDR outbreak). Among the 6,262 patient isolates included in multistate BYP-associated salmonellosis outbreaks, 2,248 (36%) were resistant, 209 (3%) were MDR, and 395 (6%) were CRR.

**Discussion:**

Although nearly half of BYP-associated salmonellosis outbreaks and one-third of outbreak isolates were resistant, CRR and MDR outbreaks and isolates were infrequent. Continued monitoring for antimicrobial resistance is warranted because of the persistence of BYP-associated salmonellosis and the potential for BYP to serve as a reservoir for resistant *Salmonella* that can spread to people.

## Introduction

In the United States, nontyphoidal *Salmonella* causes approximately 2.4 million illnesses in people each year (1). While most of these illnesses are caused by consumption of contaminated food products, an estimated 11% of cases result from contact with live animals (2). Backyard poultry (BYP) include privately-owned chickens, ducks, turkeys, geese, guinea fowl, and quail that are not marketed for commercial food production or use. BYP are associated with more salmonellosis outbreaks and outbreak-associated illnesses than contact with any other animal; during 2015–2018, there were 23 outbreaks linked to BYP versus 8 linked to other animal species (3, 4). Multistate BYP-associated salmonellosis (BYPAS) outbreaks are detected and investigated every year (5, 6).

Salmonellosis causes diarrhea, fever, and abdominal pain that usually resolves without treatment, but some infections may require treatment with antimicrobials. Antimicrobial-resistant strains are associated with more severe infections and higher rates of hospitalization (7–10). Antimicrobial resistance in *Salmonella* is worsening in the United States: during 2015–2016, 16% of all isolates were nonsusceptible to at least one antimicrobial recommended for treatment, a 40% increase compared with 2004–2014 (11, 12).

During 2015–2018, 57% of BYPAS outbreaks were resistant compared with 100% of outbreaks linked to other animals (4). BYPAS outbreak isolates were less likely to be resistant to any antimicrobial or to be multidrug resistant compared with isolates tested by CDC’s National Antimicrobial Resistance Monitoring System (NARMS); NARMS routinely tests a representative sample of isolates from all U.S. *Salmonella* infections for surveillance (4). However, due to the persistence of BYPAS outbreaks and potential to transmit resistant bacteria to people, continued monitoring is warranted. Few analyses have focused specifically on recent trends and resistance patterns among BYPAS outbreaks (13), leaving an important gap in understanding the current epidemiology and public health risk.

The objectives of this study were to characterize antimicrobial resistance patterns among BYPAS outbreaks and outbreak-associated isolates collected during 2018–2023 and to evaluate differences in demographic characteristics and clinical outcomes between patients infected with resistant versus susceptible isolates.

## Methods

### Case and outbreak characterization

Laboratory-confirmed cases of BYPAS from 2018 to 2023 were included in this analysis (14). State and local public health laboratories characterized patient isolates using pulsed-field gel electrophoresis (PFGE) for 2018 outbreaks and January–June 2019 outbreaks, and whole genome sequencing (WGS) beginning in July 2019 through 2023, along with core genome multilocus sequence typing, to assess genetic relatedness among nontyphoidal *Salmonella* isolates (5, 6, 15). Only multistate BYPAS outbreaks were analyzed, defined as those including ≥2 patients residing in ≥2 U.S. states or territories, with genetically related isolates and epidemiologic evidence linking illnesses to BYP.

We defined BYPAS as described in Stapleton et al. (6). Briefly, contact was defined as direct or indirect interaction with poultry or their environment, consumption of eggs or meat obtained from BYP, or residence with a household member who directly interacted with BYP that occurred within 7 days preceding illness onset. Questionnaire data obtained from patient interviews by local public health jurisdictions were shared with CDC in the System for Enteric Disease Response, Investigation, and Coordination (SEDRIC). When possible, multistate BYPAS outbreak patients were also asked a supplementary set of standardized BYP exposure questions; those data were shared with CDC via Epi Info™ (6).

### Antimicrobial resistance characterization

The NARMS laboratory performed antimicrobial susceptibility testing (AST) on a subset of isolates using broth microdilution (Sensititre^®^, Westlake, OH) following the manufacturer’s instructions (Thermo Fisher Scientific, Waltham, MA, USA) for 15 antimicrobials from 11 classes (Supplementary Image 1). Breakpoints and interpretive criteria established by the Clinical and Laboratory Standards Institute (CLSI) were used to determine susceptibility or resistance when available (16). We considered isolates with intermediate interpretation to ciprofloxacin to be resistant because minimum inhibitory concentrations (MICs) in the intermediate range (0.12-0.5 ug/mL) have been shown to affect clinical outcomes (16, 25). NARMS-defined breakpoints of MIC ≥32 μg/mL for resistance to azithromycin and streptomycin were used because CLSI does not have clinical breakpoints for these antimicrobials in nontyphoidal *Salmonella* (17).

AST results were not available for kanamycin, ceftiofur, trimethoprim, fosfomycin, streptomycin (during 2020–2023), and colistin (during 2018–2019) (Supplementary Image 1). WGS data on isolates in BYPAS outbreaks were screened for antimicrobial resistance determinants using ResFinder 5.0 (commit date: 02/04/2022; 90% identity, 50% gene coverage) and PointFinder (commit date: 02/10/2022), using previously published methods (18). AST results were prioritized for resistance determinations. When AST results were not available, predicted resistance from WGS was used; predicted resistance from WGS has been shown to have high concordance with phenotypic resistance from AST (19).

Isolates were considered susceptible when susceptible to all antimicrobials tested by NARMS; resistant when resistant to ≥1 antimicrobial drug; multidrug resistant (MDR) when resistant to at least one antimicrobial from ≥3 classes (4, 20); having clinically relevant resistance (CRR) when resistant to ampicillin, azithromycin, ceftriaxone, ciprofloxacin, or trimethoprim-sulfamethoxazole (21, 22); and extensively drug resistant (XDR) when resistant to all five clinically relevant antimicrobials. We defined outbreaks with any resistance, CRR, MDR, or XDR when ≥3 isolates and ≥10% of isolates met the corresponding definition of isolate-level resistance. Resistance classifications of outbreaks were not mutually exclusive.

### Data analysis

Data from SEDRIC and NARMS were linked using unique isolate identifiers. Patient ethnicity and race were analyzed and reported together. We classified patients who reported Hispanic or Latino ethnicity as Hispanic or Latino; patients who reported neither Hispanic nor Latino ethnicity, or did not report ethnicity, were categorized based on self-reported race. Patient residence was determined according to regions defined by the U.S. Census Bureau (23). Deaths among BYPAS outbreak patients were only reported to CDC if attributed to salmonellosis.

Except for age, patient variables were compared by resistance status using Chi-squared or Fisher’s exact tests (when cell counts were ≤ 5), followed by *post hoc* pairwise comparisons of proportions with Holm correction. Patient ages, annual number of outbreaks, and outbreak size by resistance status were compared using the Mann–Whitney U test. *Salmonella* serotypes that caused more than one distinct outbreak in a single year, based on genetic relatedness, were numerically labeled in the order they were detected and analyzed as distinct outbreaks (e.g., *Salmonella Enteritidis* group 1, *Salmonella Enteritidis* group 2). All analyses were performed using R Statistical Software (version 4.3.1, R Core Team 2024). Visualizations were produced using Microsoft Excel (Microsoft 365).

This activity was reviewed by CDC, deemed not research, and was conducted consistent with applicable federal law and CDC policy (see, e.g., 45 C.F.R. part 46.102(l) (2), 21 C.F.R. part 56; 42 U.S.C. §241(d); 5 U.S.C. §552a; 44 U.S.C. §3,501 et seq).

## Results

### Outbreaks

Seventy-eight multistate BYPAS outbreaks involving 6,668 patients occurred during 2018–2023. We analyzed data from the 6,262 (94%) patients whose isolates had antimicrobial resistance information. Nearly half of the outbreaks (46%, 36/78) were classified as exhibiting resistance. Among outbreaks classified as resistant, 28% were MDR (10/36) and 33% were CRR (12/36). MDR and CRR outbreaks were not mutually exclusive, with seven outbreaks classified as both MDR and CRR. None of the outbreaks were XDR.

The number of outbreaks per year ranged from 8 to 17 (median: 13 outbreaks). The median number of resistant outbreaks per year was 5 (range: 2–11) and did not differ from the median 7.5 susceptible outbreaks per year (range: 3–10, *p* = 0.8). The median annual number of MDR outbreaks was 0.5 (range: 0–6), and the median annual number of CRR outbreaks was 2 (range: 0–4, Figure 1). The median size of all BYPAS outbreaks was 46 cases (range: 6–835). The size of susceptible outbreaks (median: 43 cases, range: 6–313) did not differ from the size of resistant outbreaks (median: 51 cases, range: 7–835; *p* = 0.8), MDR outbreaks (median: 67 cases, range: 13–147; *p* = 0.5), or CRR outbreaks (median: 69 cases, range: 13–147; *p* = 0.2). The largest resistant outbreak was caused by *Salmonella* Hadar in 2020 (835 illnesses), and the largest CRR and MDR outbreaks were the same *Salmonella* Braenderup outbreak in 2019 (147 illnesses).

**Figure 1:**
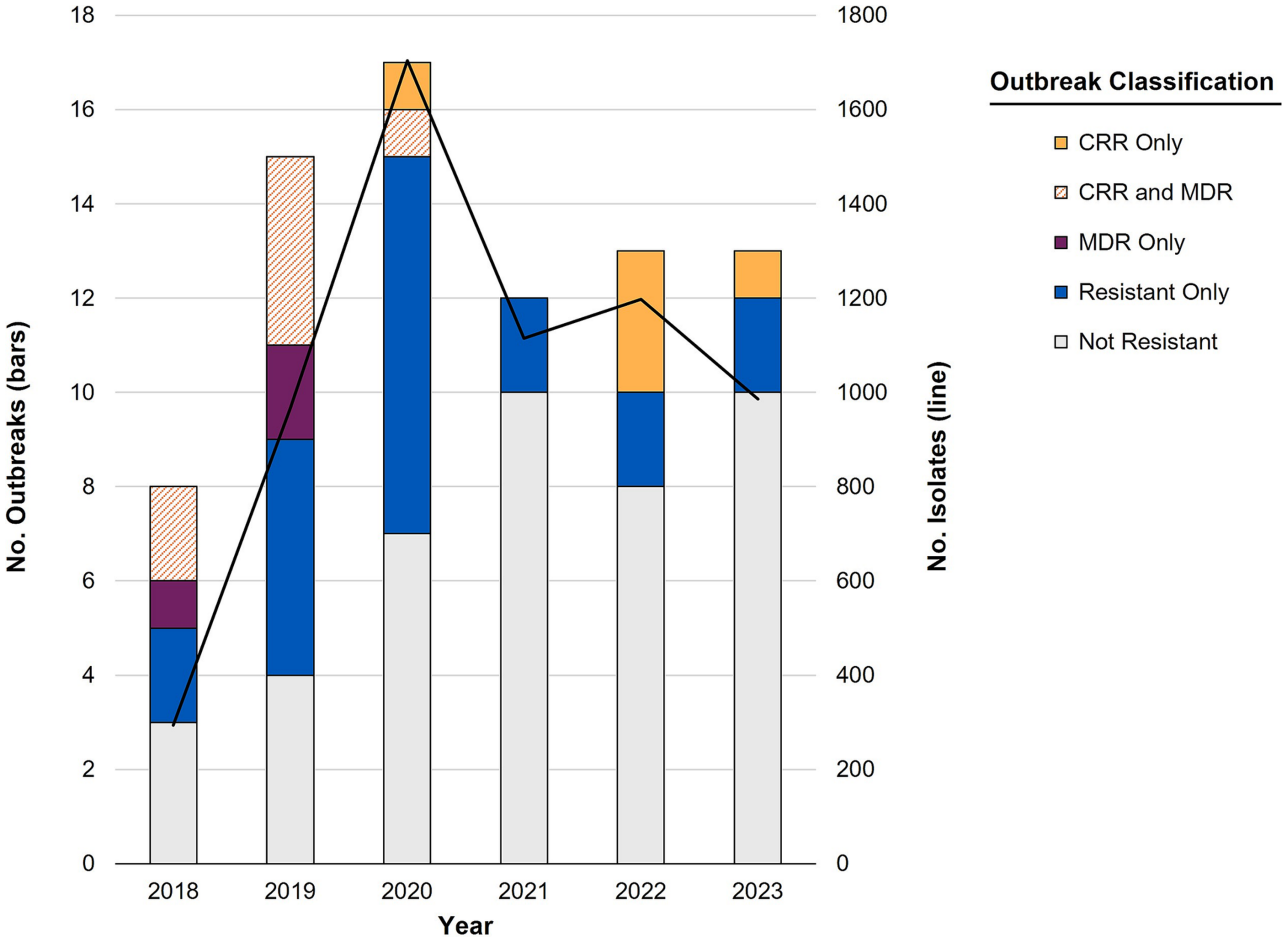
Annual count of clinical isolates (line) and multistate backyard poultry associated salmonellosis outbreaks by resistance classification (bars), 2018–2023. The figure depicts the annual distribution of the 78 multistate BYPAS outbreaks, and 6,262 isolates included in these analyses.

Twenty serotypes caused multistate BYPAS outbreaks during the study period. The most frequent serotypes causing outbreaks were *Salmonella Enteritidis* (22 outbreaks) and *Salmonella* Infantis (10 outbreaks). Among the 22 Enteritidis outbreaks, three (14%) were classified as resistant (two CRR-only outbreaks; one outbreak was both CRR and MDR, Supplementary Table 1). In the two resistant Enteritidis outbreaks that occurred in 2022, 100% of isolates in both outbreaks were ciprofloxacin nonsusceptible; in the Enteritidis outbreak in 2018, only 2% of isolates were ciprofloxacin nonsusceptible. Among the 10 Infantis outbreaks, six (60%) were classified as having any resistance; of these, two outbreaks had both CRR and MDR.

The proportion of resistant isolates belonging to outbreaks classified as resistant ranged from 13 to 100% (Supplementary Table 1). The proportion of isolates that were MDR in the 10 MDR outbreaks ranged from 10 to 92%; the proportion of CRR isolates in the 12 CRR outbreaks was 12–100%.

### Isolates

#### BYPAS patients

Among 6,262 patients, 4,014 (64%) were infected with susceptible strains of nontyphoidal *Salmonella*, and 2,248 (36%) were infected with strains that were resistant to ≥1 antimicrobial. The median patient age was 35 years (range: <1–102 years) and did not differ by resistance status (*p* = 0.6, Table 1). However, the distribution of patients’ ages differed based on whether they had a resistant or susceptible infection (*p* < 0.01). Patients aged <5 years composed a higher proportion of resistant infections (25%) than susceptible infections (21%, *p* < 0.01).

**Table 1:** Comparison of demographic, behavioral, and outcome characteristics of patients in multistate BYP-associated nontyphoidal salmonellosis outbreaks, by isolate antimicrobial susceptibility—United States, 2018–2023.^†^

Characteristic	All isolates	Susceptible	Resistant^‡^	*p*-value^§^
	*N* = 6,262	*N* = 4,014	*N* = 2,248	
Age, median (Min–Max)	35 (0–102)	34 (0–102)	37 (0–98)	0.64
Age group, *n* (%)	*N* = 6,159	*N* = 3,932	*N* = 2,227	**<0.01**
<5	1,389 (22.6)	835 (21.2)	554 (24.9)	
5–17	790 (12.8)	548 (13.9)	242 (10.9)	
18–64	2,976 (48.3)	1,914 (48.7)	1,062 (47.7)	
≥65	1,004 (16.3)	635 (16.1)	369 (16.6)	
Sex, *n* (%)	*N* = 6,085	*N* = 3,904	*N* = 2,181	0.40
Female	3,476 (57.1)	2,214 (56.7)	1,262 (57.9)	
Male	2,609 (42.9)	1,690 (43.3)	919 (42.1)	
Ethnicity and race, *n* (%)	*N* = 4,024	*N* = 2,614	*N* = 1,410	**<0.01**
Hispanic or Latino	428 (10.6)	237 (9.1)	191 (13.5)	
American Indian or Alaska Native	52 (1.3)	35 (1.3)	17 (1.2)	
Asian	38 (0.9)	27 (1.0)	11 (0.8)	
Black or African American	105 (2.6)	78 (3.0)	27 (1.9)	
Native Hawaiian or Other Pacific Islander	6 (0.1)	4 (0.2)	2 (0.1)	
White	3,336 (82.9)	2,188 (83.7)	1,148 (81.4)	
More than 1 race	36 (0.9)	31 (1.2)	5 (0.4)	
Other	23 (0.6)	14 (0.5)	9 (0.6)	
Region^¶^, *n* (%)	*N* = 6,262	*N* = 4,014	*N* = 2,248	**<0.01**
Midwest	1,982 (31.7)	1,419 (35.4)	563 (25.0)	
Northeast	1,100 (17.6)	737 (18.4)	363 (16.1)	
South	2,112 (33.7)	1,298 (32.3)	814 (36.2)	
West	1,060 (16.9)	553 (13.8)	507 (22.6)	
Hospitalized, *n* (%)	*N* = 4,384	*N* = 2,847	*N* = 1,537	**<0.01**
	1,377 (31.4)	843 (29.6)	534 (34.7)	
Died, *n* (%)	*N* = 4,118	*N* = 2,663	*N* = 1,455	0.43
	7 (0.2)	6 (0.2)	1 (0.1)	
Specimen type, *n* (%)	*N* = 6,180	N = 3,968	*N* = 2,212	0.22
Blood	351 (5.7)	242 (6.1)	109 (4.9)	
Stool	5,351 (86.6)	3,425 (86.3)	1,926 (87.1)	
Urine	431 (7.0)	269 (6.8)	162 (7.3)	
Other	47 (0.8)	32 (0.8)	15 (0.7)	

† BYP, backyard poultry. Patients with missing or “unknown” epidemiologic information were excluded from the analysis; denominators reflect cases with available data (*N*).

‡ Refers to isolates categorized as resistant (see Methods).

§ Bold print indicates a *p*-value less than *α* = 0.05.

¶ Eight patients who resided in Puerto Rico are included in the denominator (*N*) but are not included in the table values.

Most patients involved in multistate BYPAS outbreaks were female (57%) and White (83%, Table 1). A higher proportion of infections in Hispanic or Latino patients were resistant (14%) than susceptible (9%, *p* < 0.01). Approximately one-third of all patients lived in the Midwest (32%) and one-third in the South (34%). The distribution of patients’ region of residence differed by resistance status (*p* < 0.01). Compared with patients infected with susceptible strains, a higher proportion of resistant infections occurred in patients living in the West (23% of resistant infections versus 14% of susceptible infections, *p* < 0.01) and a lower proportion of resistant infections occurred in the Midwest (25% of resistant infections versus 35% of susceptible infections, *p* < 0.01).

Nearly one third (31%) of all patients were hospitalized. A greater proportion of patients with resistant infections were hospitalized (35%) compared with patients with non-resistant infections (30%, *p* < 0.01). Seven patients (0.2%) died from BYPAS infections. The specimen type (i.e., blood, stool, urine, or other type) was not associated with the presence of resistance (*p* = 0.2).

#### Resistant isolates

Most (64%) multistate BYPAS isolates were susceptible. Of the 2,248 resistant isolates, 209 (9%) were MDR, 395 (18%) were CRR, and 123 (6% of 2,248 resistant isolates) were both MDR and CRR.

Serotype Hadar had the greatest number of isolates with any resistance (*n* = 1,442, 99%), followed by *Salmonella Enteritidis* (*n* = 228, 10%) and Infantis (*n* = 158, 17%) (Table 2). Other serotypes with a high proportion of resistant isolates were *Salmonella* Manhattan (94%), Agona (93%), Montevideo (92%), and Alachua (92%).

**Table 2:** Resistance determinations for patients included in multistate BYP-associated nontyphoidal salmonellosis outbreaks by *Salmonella* serotype—United States, 2018–2023.^†^

*Salmonella* serotype	Cases (*N* = 6,262) *n* (column %)	No. of outbreaks	Susceptible isolates *n* (row %)	Resistant isolates *n* (row %)	MDR isolates^‡^ *n* (row %)	CRR isolates^§^ *n* (row %)
Agona	87 (1.4)	2	6 (6.9)	81 (93.1)	0 (0)	0 (0)
Alachua	13 (0.2)	1	1 (7.7)	12 (92.3)	3 (23.1)	3 (23.1)
Altona	7 (0.1)	1	3 (42.9)	4 (57.1)	1 (14.3)	0 (0)
Anatum	108 (1.7)	2	84 (77.8)	24 (22.2)	20 (18.5)	20 (18.5)
Braenderup	370 (5.9)	5	288 (77.8)	82 (22.2)	24 (6.5)	28 (7.6)
Enteritidis	2,193 (35.0)	22	1965 (89.6)	228 (10.4)	12 (0.5)	220 (10.0)
Hadar	1,461 (23.3)	3	19 (1.3)	1,442 (98.7)	36 (2.5)	14 (1.0)
I 4,[5],12:i:-	66 (1.1)	2	46 (69.7)	20 (30.3)	2 (3.0)	5 (7.6)
Indiana	77 (1.2)	4	76 (98.7)	1 (1.3)	1 (1.3)	1 (1.3)
Infantis	938 (15.0)	10	780 (83.2)	158 (16.8)	52 (5.5)	43 (4.6)
Litchfield	9 (0.1)	1	3 (33.3)	6 (66.7)	2 (22.2)	1 (11.1)
Manhattan	36 (0.6)	1	2 (5.6)	34 (94.4)	33 (91.7)	0 (0)
Mbandaka	259 (4.1)	6	241 (93.1)	18 (6.9)	4 (1.5)	5 (1.9)
Montevideo	66 (1.1)	3	5 (7.6)	61 (92.4)	6 (9.1)	3 (4.5)
Muenchen	56 (0.9)	3	55 (98.2)	1 (1.8)	0 (0)	0 (0)
Newport	96 (1.5)	2	80 (83.3)	16 (16.7)	9 (9.4)	7 (7.3)
Oranienburg	9 (0.1)	1	9 (100)	0 (0)	0 (0)	0 (0)
Senftenberg	28 (0.4)	1	28 (100)	0 (0)	0 (0)	0 (0)
Thompson	22 (0.4)	1	20 (90.9)	2 (9.1)	1 (4.5)	0 (0)
Typhimurium	361 (5.8)	7	303 (83.9)	58 (16.1)	3 (0.8)	45 (12.5)

† BYP, backyard poultry. Percentages in ‘Cases’ column represent the proportion of total cases; percentages in other columns represent the proportion of resistant isolates within each serotype.

‡ MDR, multidrug resistant.

§ CRR, clinically relevant resistant. Clinically relevant antimicrobials include ampicillin, azithromycin, ceftriaxone, ciprofloxacin, and trimethoprim-sulfamethoxazole.

**Figure 2:**
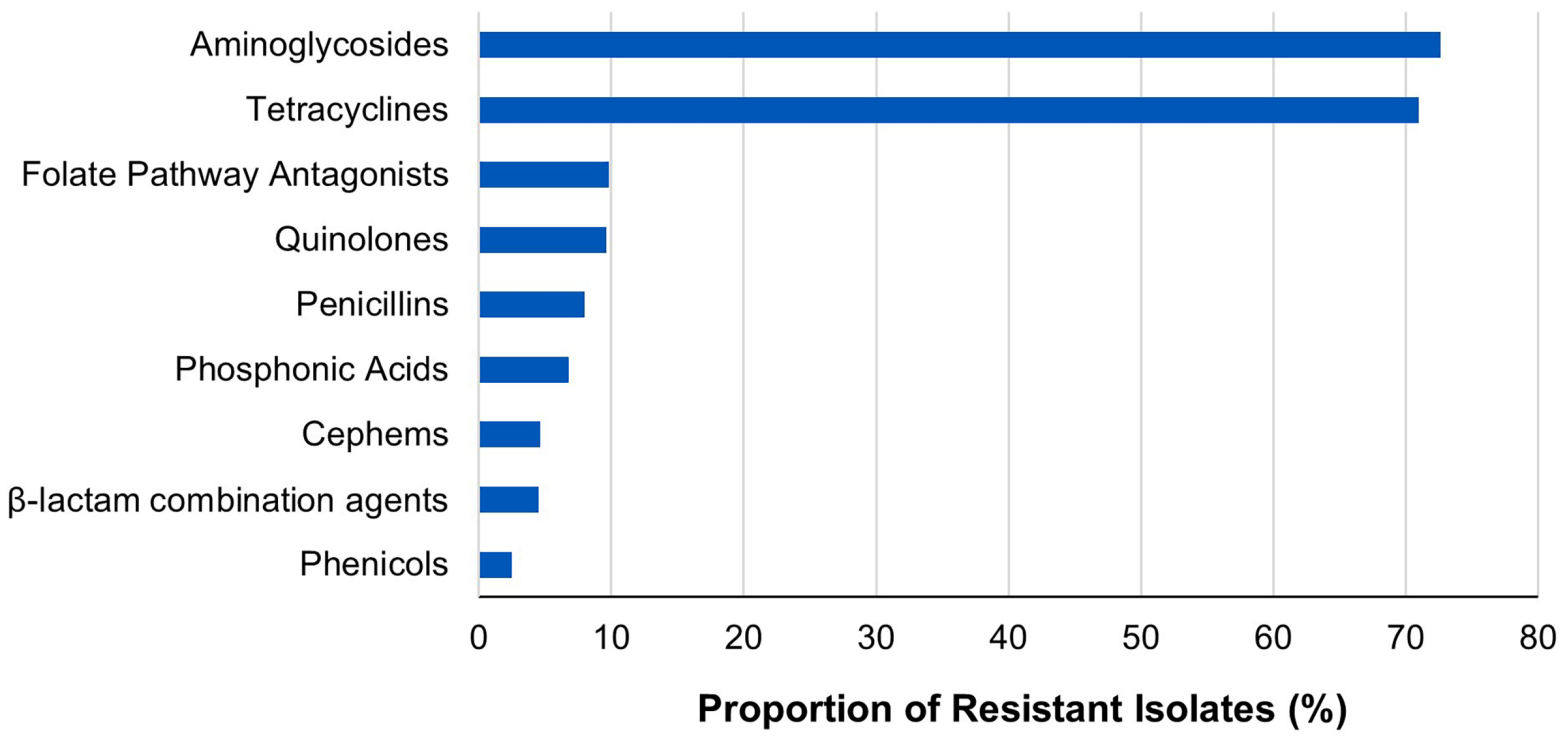
Distribution of resistance by antimicrobial class among BYPAS isolates with any resistance (*N* = 2,248). No isolates were resistant to antimicrobials from macrolide or penem classes. Representative antimicrobial drugs for each class can be found in Supplementary Table 2.

Resistance to the aminoglycosides (*n* = 1,631, 73%) or tetracyclines (*n* = 1,596, 71%) was most frequent among 2,248 isolates classified as resistant (Figure 2; Supplementary Table 2). None of the isolates were resistant to macrolides or penems. Isolate resistance to antimicrobial classes and specific antimicrobials can be found in Supplementary Table 2.

#### Multi-drug resistant (MDR) isolates

Among the 209 MDR isolates, the most frequent serotypes were Infantis (*n* = 52, 25%), Hadar (*n* = 36, 17%), and Manhattan (*n* = 33, 16%). Serotypes with the highest relative proportion of MDR isolates were *Salmonella* Manhattan (92%), *Salmonella* Alachua (23%), and *Salmonella* Litchfield (22%) (Table 2). MDR isolates were resistant to penicillins (*n* = 122, 58%), aminoglycosides (*n* = 120, 57%), folate pathway antagonists (*n* = 114, 55%), cephems (*n* = 103, 49%), *β*-lactam combination agents (*n* = 101, 48%), tetracyclines (*n* = 75, 36%), phenicols (*n* = 56, 27%), and quinolones (*n* = 2, 1%). The most frequent MDR combination was ampicillin, amoxicillin-clavulanic acid, ceftriaxone, ceftiofur, and cefoxitin (*n* = 81, 39%).

#### Clinically relevant resistance

**Figure 3:**
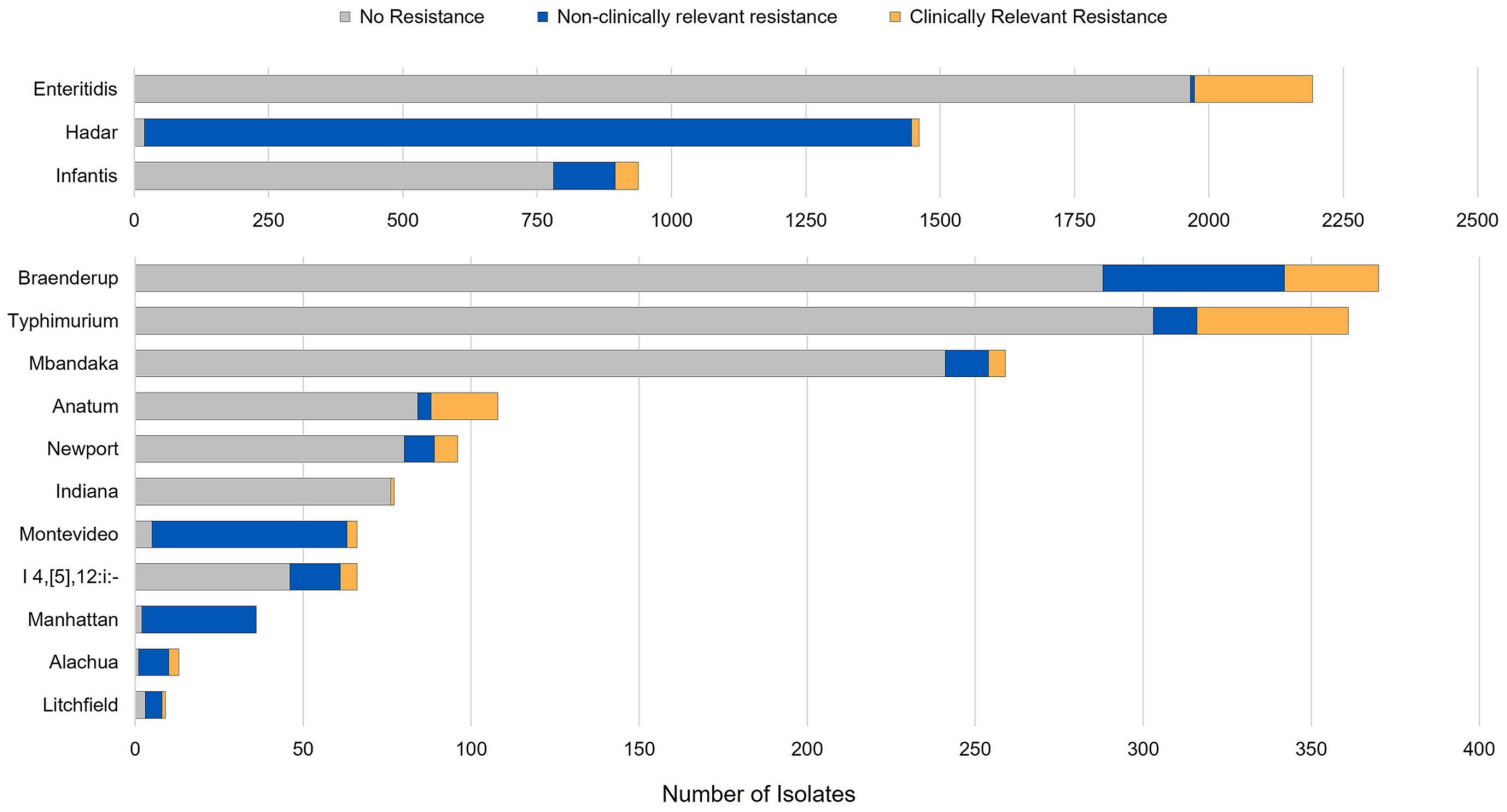
Comparison of resistance status by clinical relevance among BYPAS isolates by serotype. Serotypes Enteritidis, Hadar, and Infantis are depicted on the top panel, all other serotypes are depicted on the bottom panel (note differing scales). Serotypes Agona, Altona, Muenchen, Oranienburg, Senftenberg, and Thompson are not shown because none had isolates with CRR.

Among the 395 CRR isolates, 220 (56%) were *Salmonella Enteritidis*, 45 (11%) were Typhimurium, and 43 (11%) were Infantis. The serotypes with the highest proportion of CRR isolates were Alachua (23%), Anatum (19%), and Typhimurium (13%) (Table 2 and Figure 3).

Two hundred and fifteen of the 395 CRR isolates (54%) were nonsusceptible to ciprofloxacin, 180 (46%) were resistant to ampicillin, 103 (26%) were resistant to ceftriaxone, and 54 (14%) were resistant to trimethoprim-sulfamethoxazole (Table 3). None were resistant to azithromycin. One hundred and fifty-five (39%) isolates were resistant to ≥2 clinically relevant antimicrobials, and one (0.3%) *Salmonella Typhimurium* isolate was resistant to three clinically relevant antimicrobials: ampicillin, ciprofloxacin, and trimethoprim-sulfamethoxazole (Supplementary Table 3).

**Table 3:** Serotypes represented in backyard poultry-associated outbreaks of *Salmonella* illnesses by resistance to clinically relevant antimicrobials—United States, 2018–2023.^†^

*Salmonella* serotype^‡^	No. isolates with any resistance, *n*	Ampicillin	Ceftriaxone	Ciprofloxacin	Trimethoprim-Sulfamethoxazole
		*n* (% of resistant isolates)^§^			
Agona	81	0 (0)	0 (0)	0 (0)	0 (0)
Alachua	12	3 (25.0)	1 (8.3)	0 (0)	0 (0)
Altona	4	0 (0)	0 (0)	0 (0)	0 (0)
Anatum	24	19 (79.2)	19 (79.2)	1 (4.2)	0 (0)
Braenderup	82	27 (32.9)	23 (28.0)	0 (0)	2 (2.4)
Enteritidis	228	13 (5.7)	0 (0)	207 (90.8)	0 (0)
Hadar	1,442	11 (0.8)	7 (0.5)	3 (0.2)	4 (0.3)
I 4,[5],12:i:-	20	5 (25.0)	0 (0)	0 (0)	4 (20.0)
Indiana	1	1 (100)	1 (100)	0 (0)	0 (0)
Infantis	158	41 (25.9)	37 (23.4)	2 (1.3)	0 (0)
Litchfield	6	1 (16.7)	1 (16.7)	0 (0)	0 (0)
Manhattan	34	0 (0)	0 (0)	0 (0)	0 (0)
Mbandaka	18	4 (22.2)	4 (22.2)	1 (5.6)	0 (0)
Montevideo	61	3 (4.9)	3 (4.9)	0 (0)	0 (0)
Muenchen	1	0 (0)	0 (0)	0 (0)	0 (0)
Newport	16	7 (43.8)	7 (43.8)	0 (0)	0 (0)
Thompson	2	0 (0)	0 (0)	0 (0)	0 (0)
Typhimurium	58	45 (77.6)	0 (0)	1 (1.7)	44 (75.9)
Total	2,248	180	103	215	54

† Clinically relevant antimicrobials for nontyphoidal salmonellosis include ampicillin, azithromycin, ceftriaxone, ciprofloxacin, and trimethoprim-sulfamethoxazole. None of the isolates in this study were resistant to azithromycin.

‡ Salmonella serotypes with no resistant isolates (Oranienburg and Senftenberg) were excluded from the table.

§ Isolates could be resistant to more than one clinically relevant antimicrobial.

Nearly all (91%) of the 228 resistant *Salmonella Enteritidis* were nonsusceptible to ciprofloxacin. Further, 79% of the resistant *Salmonella* Anatum isolates were resistant to both ampicillin and ceftriaxone, and most resistant *Salmonella Typhimurium* isolates were resistant to ampicillin (78%) and trimethoprim-sulfamethoxazole (76%) (Table 3).

## Discussion

During 2018–2023, there were 78 multistate BYPAS outbreaks involving 6,668 patients (a median of 13 outbreaks per year). We classified nearly half the outbreaks as resistant. Among those, one quarter of outbreaks demonstrated resistance to clinically relevant antimicrobials, and one third were MDR. A previous study by Frey et al. (4) similarly assessed antimicrobial resistance in animal contact-associated salmonellosis outbreaks and reported 23 multistate BYPAS outbreaks occurring during 2015–mid-2018 (approximately 6.5 outbreaks per year). In that study, 57% of outbreaks (*n* = 13) were classified as resistant using criteria identical to this study. This suggests that while the annual number of multistate BYPAS outbreaks has increased, the proportion of BYPAS outbreaks that were resistant has remained relatively stable and might be decreasing (6). Additionally, the proportion of resistant isolates in outbreaks classified as resistant was variable (13–100%). Although greater genetic diversity is expected in outbreaks linked to animal contact (24), the reasons for the proportionate heterogeneity of AR among isolates within BYPAS outbreaks warrants further study.

Almost two-thirds of isolates included in this study were susceptible, and among the remaining one third of isolates that were resistant, less than 20% were CRR; even fewer (less than 10%) were MDR and none were XDR. Two thirds of the resistant isolates in our study were *Salmonella* Hadar, which was the second most common serotype causing multistate BYPAS outbreaks and outbreak-associated illnesses in our study and a preceding study (6). Nearly all (~99%) Hadar isolates were resistant to at least one antimicrobial, but few of the resistant isolates were MDR or CRR (<2.5%). Regardless, our results align with other studies that show patients with resistant infections have higher hospitalization rates than patients with susceptible infections, suggesting the presence of antimicrobial resistance genes, even if not clinically relevant, may interact with other microbial mechanisms that worsen clinical outcomes (7–10).

Clinically relevant antimicrobials are those recommended as first-line therapeutics to treat salmonellosis in people when antibiotics are indicated (21, 22). If prescribed empirically or before AST results are available, patients with CRR *Salmonella* infections could experience treatment failure and worsening conditions. In our study, over half of CRR isolates were nonsusceptible to ciprofloxacin; this is concerning because even small increases in quinolone MICs are associated with worse clinical outcomes (25, 26). Still, the overall proportion of BYPAS outbreak isolates that had CRR was low (~6% of all isolates). This is less than the estimated 13% of *Salmonella* isolates that were CRR among clinical isolates collected during 2004–2016 (12), and less than the 22% of isolates that were CRR from retail meat isolates (i.e., poultry, beef, and pork) collected during 2016–2019 (27). However, CRR in *Salmonella* has been increasing over time, primarily driven by ciprofloxacin nonsusceptibility in *Salmonella Enteritidis* (12, 28). U.S. surveillance indicates that the predominant mechanism for decreased susceptibility to ciprofloxacin in *Salmonella Enteritidis* is a mutation in the *gyrA* gene. Closely related strains of Enteritidis carrying this mutation have been linked to human illnesses associated with consumption of commercial poultry products (e.g., meat and eggs) and contact with BYP, and have also been isolated from retail chicken meat and chickens at slaughter (28).

Like *Salmonella Enteritidis*, closely related strains of serotype Hadar with resistance to streptomycin and tetracycline have also been linked to both BYP and the commercial poultry food industry (29). Examples such as these highlight the intersection between the BYP industry and poultry that are produced in large flocks for commercial eggs and meats; BYP hatcheries sometimes source certain types of birds from suppliers who also provide eggs and birds to food poultry producers (30). This practice could result in the spread of resistance mechanisms across industries. The connections between the BYP and food poultry industries are complex, and exchange of resistant strains of *Salmonella* between the two sectors is not certain. For example, a MDR strain of *Salmonella* Infantis that has been found in retail poultry products and in human clinical isolates has not been linked to BYP (31–33). Investigation of *Salmonella* strains and resistance mechanisms that are found across different types of commodities might provide clues for transmission and prevention strategies. Collaboration between public health, animal health, and poultry industry sectors is needed to identify practices that contribute to spread and persistence of resistant strains.

This analysis was subject to at least the following limitations. First, due to challenges in collecting data during outbreak investigations, missing data were observed and excluded for some demographic and exposure variables. Second, outbreak case definitions were based on PFGE during the first 18 months of the study period and on WGS thereafter; however, the impact of this transition on case detection and resistance assessment is tempered by the fact that most isolates still underwent WGS in these early study years. Third, salmonellosis is a self-limiting illness, and many ill people do not seek healthcare for their infection or have a diagnostic test performed to confirm the cause of their illness. Therefore, the true number of cases involved in these outbreaks is estimated to be under-diagnosed at approximately 38 to 40 infections for every one detected (1). Fourth, single-state outbreaks were not included in this analysis. Our results may not be generalizable to more localized outbreaks. Finally, due to the increased reliance on predicted resistance from WGS, not all isolates were phenotypically tested for all antimicrobials (which is considered the gold standard). However, due to the high correlation between predicted resistance and phenotypic resistance for most study drugs (19), resistance methods likely had a limited impact on our findings.

Salmonellosis outbreaks linked to contact with BYP continue to occur annually in the United States (5, 6, 34). Surveillance for enteric pathogens associated with animal contact is important to understand antimicrobial resistance diversity, which tends to be greater in strains causing outbreaks from contact with live animals compared with strains that contaminate food products (4). Public health investigations of BYPAS outbreaks and monitoring of antimicrobial resistance in BYPAS isolates remain important for understanding the emergence and transmission of antimicrobial resistant *Salmonella*.

## Data Availability

The data analyzed in this study is subject to the following licenses/restrictions: The data that support the findings of this study are available from the corresponding author upon reasonable request. Requests to access these datasets should be directed to hwj7@cdc.gov.
