# Supplementary material for: Antimicrobial resistance in nontyphoidal Salmonella associated with multistate outbreaks linked to backyard poultry, United States, 2018–2023
Source: Front Public Health. 2026 Jun 12;14:1854943. doi: 10.3389/fpubh.2026.1854943 (PMC13303768; doi:10.3389/fpubh.2026.1854943)
Supplement: Supplementary file 1 [file Image_1.pdf]

Supplementary Figure 1: Antimicrobial test by NARMS using antimicrobial susceptibility testing (AST) and antimicrobial resistance prediction using whole genome sequencing (WGS) by year, 2018–2023. Resistance in a single isolate could have been determined by AST, WGS, or both. AST results were preferentially used to determine resistance. If AST results were not available, predicted resistance based on WGS was used.

Antimicrobial Class	Antimicrobial Drug	2018	2019	2020	2021	2022	2023
Aminoglycosides	Streptomycin	AST and WGS	AST and WGS	WGS only	WGS only	WGS only	WGS only
	Gentamicin	AST and WGS	AST and WGS	AST and WGS	AST and WGS	AST and WGS	AST and WGS
	Kanamycin	WGS only	WGS only	WGS only	WGS only	WGS only	WGS only
β–lactam combination agents	Amoxicillin-Clavulanic Acid	AST and WGS	AST and WGS	AST and WGS	AST and WGS	AST and WGS	AST and WGS
Cephems	Ceftriaxone	AST and WGS	AST and WGS	AST and WGS	AST and WGS	AST and WGS	AST and WGS
	Ceftiofur	WGS only	WGS only	WGS only	WGS only	WGS only	WGS only
	Cefoxitin	AST and WGS	AST and WGS	AST and WGS	AST and WGS	AST and WGS	AST and WGS
Folate Pathway Antagonists	Trimethoprim-Sulfamethoxazole	AST and WGS	AST and WGS	AST and WGS	AST and WGS	AST and WGS	AST and WGS
	Sulfisoxazole	AST and WGS	AST and WGS	AST and WGS	AST and WGS	AST and WGS	AST and WGS
	Trimethoprim	WGS only	WGS only	WGS only	WGS only	WGS only	WGS only
Fosfomycin	Fosfomycin	WGS only	WGS only	WGS only	WGS only	WGS only	WGS only
Penicillins	Ampicillin	AST and WGS	AST and WGS	AST and WGS	AST and WGS	AST and WGS	AST and WGS
Phenicols	Chloramphenicol	AST and WGS	AST and WGS	AST and WGS	AST and WGS	AST and WGS	AST and WGS
Polymixins	Colistin	WGS only	WGS only	AST and WGS	AST and WGS	AST and WGS	AST and WGS
Quinolones	Ciprofloxacin	AST and WGS	AST and WGS	AST and WGS	AST and WGS	AST and WGS	AST and WGS
	Nalidixic Acid	AST and WGS	AST and WGS	AST and WGS	AST and WGS	AST and WGS	AST and WGS

Key:
- WGS only
- AST and WGS
