# Supplementary material for: Antimicrobial resistance in nontyphoidal Salmonella associated with multistate outbreaks linked to backyard poultry, United States, 2018–2023
Source: Front Public Health. 2026 Jun 12;14:1854943. doi: 10.3389/fpubh.2026.1854943 (PMC13303768; doi:10.3389/fpubh.2026.1854943)
Supplement: Supplementary file 2 [file Table_1.DOCX]

**Supplementary Table 1.** Antimicrobial resistance patterns for outbreaks of nontyphoidal salmonellosis associated with backyard poultry exposure, detected by antimicrobial susceptibility testing or whole genome sequencing, 2018-2023.

| **Year** | **Serotype** | **Outbreak Resistance Categorization**^a^ | **Resistance Pattern**^b,c^ | **Isolates^d^** |
| --- | --- | --- | --- | --- |
| 2018 | Enteritidis 1 | Not resistant | None | 52/52 (100) |
| 2018 | Enteritidis 2 | CRR and MDR | None | 53/66 (80.3) |
|  |  |  | A | 1/66 (1.5) |
|  |  |  | ASSuT | 11/66 (16.7) |
|  |  |  | Cip(I/R)Nal | 1/66 (1.5) |
| 2018 | Indiana | Not resistant | None | 15/15 (100) |
| 2018 | Infantis | CRR and MDR | None | 55/67 (82.1) |
|  |  |  | AAuCxTioFox | 8/67 (11.9) |
|  |  |  | S | 1/67 (1.5) |
|  |  |  | SSu | 1/67 (1.5) |
|  |  |  | SSuGen | 2/67 (3.0) |
| 2018 | Litchfield | Resistant | None | 3/9 (33.3) |
|  |  |  | ASTAuCxTioFox | 1/9 (11.1) |
|  |  |  | SSuTGen | 1/9 (11.1) |
|  |  |  | ST | 3/9 (33.3) |
|  |  |  | T | 1/9 (11.1) |
| 2018 | Montevideo 1 | MDR only | None | 1/38 (2.6) |
|  |  |  | AAuCxTioFoxFos | 2/38 (5.3) |
|  |  |  | ASSuTAuCxTioFoxGenFos | 1/38 (2.6) |
|  |  |  | Fos | 33/38 (86.8) |
|  |  |  | SSuTGenFos | 1/38 (2.6) |
| 2018 | Montevideo 2 | Resistant | None | 2/19 (10.5) |
|  |  |  | Fos | 15/19 (78.9) |
|  |  |  | SSuGenFos | 2/19 (10.5) |
| 2018 | Senftenberg | Not resistant | None | 28/28 (100) |
| 2019 | Agona | Resistant | None | 6/50 (12.0) |
|  |  |  | Fos | 44/50 (88.0) |
| 2019 | Alachua | CRR and MDR | None | 1/13 (7.7) |
|  |  |  | A | 1/13 (7.7) |
|  |  |  | AAuCxFox | 1/13 (7.7) |
|  |  |  | ATFos | 1/13 (7.7) |
|  |  |  | Fos | 8/13 (61.5) |
|  |  |  | SSuGenFos | 1/13 (7.7) |
| 2019 | Altona | Resistant | None | 3/7 (42.9) |
|  |  |  | CSSu | 1/7 (14.3) |
|  |  |  | T | 1/7 (14.3) |
|  |  |  | TKan | 2/7 (28.6) |
| 2019 | Anatum | CRR and MDR | None | 55/68 (80.9) |
|  |  |  | AAuCxTioFox | 9/68 (13.2) |
|  |  |  | CSSu | 1/68 (1.5) |
|  |  |  | Cip(I/R)Nal | 1/68 (1.5) |
|  |  |  | T | 2/68 (2.9) |
| 2019 | Braenderup 1 | CRR and MDR | None | 118/147 (80.3) |
|  |  |  | A | 1/147 (0.7) |
|  |  |  | AAuCxTioFox | 15/147 (10.2) |
|  |  |  | ASuCotTmp | 1/147 (0.7) |
|  |  |  | SSuCotTmp | 1/147 (0.7) |
|  |  |  | SSuGen | 2/147 (1.4) |
|  |  |  | T | 9/147 (6.1) |
| 2019 | Braenderup 2 | Resistant | None | 7/12 (58.3) |
|  |  |  | SSuGen | 2/12 (16.7) |
|  |  |  | T | 3/12 (25.0) |
| 2019 | Enteritidis 1 | Not resistant | None | 254/255 (99.6) |
|  |  |  | T | 1/255 (0.4) |
| 2019 | Enteritidis 2 | Not resistant | None | 139/139 (100) |
| 2019 | Infantis 1 | CRR and MDR | None | 74/119 (62.2) |
|  |  |  | A | 2/119 (1.7) |
|  |  |  | AAuCxTioFox | 11/119 (9.2) |
|  |  |  | ASSuAuCxTioFoxGen | 2/119 (1.7) |
|  |  |  | ASSuTAuCxTioFoxGen | 1/119 (0.8) |
|  |  |  | CSSu | 2/119 (1.7) |
|  |  |  | CSSuT | 1/119 (0.8) |
|  |  |  | Kan | 3/119 (2.5) |
|  |  |  | SGen | 1/119 (0.8) |
|  |  |  | SSuGen | 9/119 (7.6) |
|  |  |  | SSuTGen | 2/119 (1.7) |
|  |  |  | Su | 1/119 (0.8) |
|  |  |  | SuKan | 1/119 (0.8) |
|  |  |  | SuT | 1/119 (0.8) |
|  |  |  | SuTKan | 1/119 (0.8) |
|  |  |  | T | 3/119 (2.5) |
|  |  |  | TKan | 4/119 (3.4) |
| 2019 | Infantis 2 | Resistant | None | 10/19 (52.6) |
|  |  |  | Kan | 3/19 (15.8) |
|  |  |  | SSuGen | 3/19 (15.8) |
|  |  |  | TKan | 3/19 (15.8) |
| 2019 | Manhattan | MDR only | None | 2/36 (5.6) |
|  |  |  | CSSu | 33/36 (91.7) |
|  |  |  | CSu | 1/36 (2.8) |
| 2019 | Montevideo | Resistant | None | 2/9 (22.2) |
|  |  |  | Fos | 7/9 (77.8) |
| 2019 | Muenchen | Not resistant | None | 15/15 (100) |
| 2019 | Newport | MDR only | None | 56/68 (82.4) |
|  |  |  | AAuCxTioFox | 4/68 (5.9) |
|  |  |  | ASSuAuCxTioFoxGen | 1/68 (1.5) |
|  |  |  | ASuTCxTio | 1/68 (1.5) |
|  |  |  | CSSu | 1/68 (1.5) |
|  |  |  | SSuTGen | 1/68 (1.5) |
|  |  |  | T | 4/68 (5.9) |
| 2019 | Oranienburg | Not resistant | None | 9/9 (100) |
| 2020 | Agona | Resistant | Fos | 37/37 (100) |
| 2020 | Anatum | CRR and MDR | None | 29/40 (72.5) |
|  |  |  | AAuCxTioFox | 10/40 (25.0) |
|  |  |  | Kan | 1/40 (2.5) |
| 2020 | Braenderup | Resistant | None | 42/54 (77.8) |
|  |  |  | ASuTCxTio | 1/54 (1.9) |
|  |  |  | CSSuT | 1/54 (1.9) |
|  |  |  | Kan | 2/54 (3.7) |
|  |  |  | T | 7/54 (13.0) |
|  |  |  | TKan | 1/54 (1.9) |
| 2020 | Enteritidis 1 | Not resistant | None | 131/132 (99.2) |
|  |  |  | Cip(I/R) | 1/132 (0.8) |
| 2020 | Enteritidis 2 | Not resistant | None | 34/34 (100) |
| 2020 | Enteritidis 3 | Not resistant | None | 155/155 (100) |
| 2020 | Enteritidis 4 | Not resistant | None | 65/66 (98.5) |
|  |  |  | ACT | 1/66 (1.5) |
| 2020 | Hadar | Resistant | None | 8/835 (1.0) |
|  |  |  | ASSuTCotTmp | 2/835 (0.2) |
|  |  |  | ASTAuCxTioFox | 7/835 (0.8) |
|  |  |  | CSSuT | 9/835 (1.1) |
|  |  |  | S | 6/835 (0.7) |
|  |  |  | SCip(I/R) | 1/835 (0.1) |
|  |  |  | SSuT | 2/835 (0.2) |
|  |  |  | SSuTGen | 11/835 (1.3) |
|  |  |  | ST | 784/835 (93.9) |
|  |  |  | STCip(I/R) | 1/835 (0.1) |
|  |  |  | STGen | 3/835 (0.4) |
|  |  |  | STKan | 1/835 (0.1) |
| 2020 | Infantis 1 | Not resistant | None | 73/80 (91.3) |
|  |  |  | AAuCxTioFox | 2/80 (2.5) |
|  |  |  | Kan | 1/80 (1.3) |
|  |  |  | SSuGen | 1/80 (1.3) |
|  |  |  | SSuTGen | 1/80 (1.3) |
|  |  |  | SuT | 1/80 (1.3) |
|  |  |  | T | 1/80 (1.3) |
| 2020 | Infantis 2 | Resistant | None | 24/32 (75.0) |
|  |  |  | AAuCxTioFox | 2/32 (6.3) |
|  |  |  | ASSuTAuCxTioFoxGen | 1/32 (3.1) |
|  |  |  | Kan | 1/32 (3.1) |
|  |  |  | S | 3/32 (9.4) |
|  |  |  | T | 1/32 (3.1) |
| 2020 | Mbandaka | Resistant | None | 33/38 (86.8) |
|  |  |  | AAuCxTioFox | 3/38 (7.9) |
|  |  |  | T | 1/38 (2.6) |
|  |  |  | TKan | 1/38 (2.6) |
| 2020 | Muenchen | Not resistant | None | 26/26 (100) |
| 2020 | Newport | Resistant | None | 24/28 (85.7) |
|  |  |  | AAuCxTioFox | 1/28 (3.6) |
|  |  |  | SSuGen | 2/28 (7.1) |
|  |  |  | T | 1/28 (3.6) |
| 2020 | Thompson | Not resistant | None | 20/22 (90.9) |
|  |  |  | SSuTGen | 1/22 (4.5) |
|  |  |  | T | 1/22 (4.5) |
| 2020 | Typhimurium 1 | CRR only | None | 5/28 (17.9) |
|  |  |  | ASuCip(I/R)CotTmp | 1/28 (3.6) |
|  |  |  | ASuCotTmp | 22/28 (78.6) |
| 2020 | Typhimurium 2 | Resistant | None | 37/43 (86.0) |
|  |  |  | ASuCotTmp | 3/43 (7.0) |
|  |  |  | SSuGen | 1/43 (2.3) |
|  |  |  | SSuTGen | 2/43 (4.7) |
| 2020 | I 4,[5],12:i:- | Resistant | None | 46/54 (85.2) |
|  |  |  | A | 1/54 (1.9) |
|  |  |  | ASuCotTmp | 4/54 (7.4) |
|  |  |  | CSSuT | 2/54 (3.7) |
|  |  |  | SSuGen | 1/54 (1.9) |
| 2021 | Enteritidis 1 | Not resistant | None | 129/130 (99.2) |
|  |  |  | STGen | 1/130 (0.8) |
| 2021 | Enteritidis 2 | Not resistant | None | 91/92 (98.9) |
|  |  |  | T | 1/92 (1.1) |
| 2021 | Enteritidis 3 | Not resistant | None | 21/21 (100) |
| 2021 | Enteritidis 4 | Not resistant | None | 49/49 (100) |
| 2021 | Enteritidis 5 | Not resistant | None | 26/27 (96.3) |
|  |  |  | Col | 1/27 (3.7) |
| 2021 | Enteritidis 6 | Not resistant | None | 38/39 (97.4) |
|  |  |  | Cip(I/R)Tmp | 1/39 (2.6) |
| 2021 | Hadar | Resistant | None | 7/359 (1.9) |
|  |  |  | ASSuTCotTmp | 2/359 (0.6) |
|  |  |  | S | 3/359 (0.8) |
|  |  |  | SSuTGen | 1/359 (0.3) |
|  |  |  | ST | 345/359 (96.1) |
|  |  |  | T | 1/359 (0.3) |
| 2021 | Indiana | Not resistant | None | 8/8 (100) |
| 2021 | Infantis 1 | Not resistant | None | 256/283 (90.5) |
|  |  |  | A | 1/283 (0.4) |
|  |  |  | AAuCxTioFox | 5/283 (1.8) |
|  |  |  | ASSu | 1/283 (0.4) |
|  |  |  | CSSu | 2/283 (0.7) |
|  |  |  | CSSuT | 1/283 (0.4) |
|  |  |  | Cip(I/R) | 2/283 (0.7) |
|  |  |  | Gen | 1/283 (0.4) |
|  |  |  | Kan | 3/283 (1.1) |
|  |  |  | SSuGen | 4/283 (1.4) |
|  |  |  | SSuTGen | 2/283 (0.7) |
|  |  |  | STGen | 1/283 (0.4) |
|  |  |  | Su | 1/283 (0.4) |
|  |  |  | T | 3/283 (1.1) |
| 2021 | Infantis 2 | Resistant | None | 38/52 (73.1) |
|  |  |  | AAuCxTioFox | 1/52 (1.9) |
|  |  |  | SSuGen | 10/52 (19.2) |
|  |  |  | STGen | 1/52 (1.9) |
|  |  |  | TKan | 2/52 (3.8) |
| 2021 | Mbandaka | Not resistant | None | 39/40 (97.5) |
|  |  |  | STGen | 1/40 (2.5) |
| 2021 | Muenchen | Not resistant | None | 14/15 (93.3) |
|  |  |  | T | 1/15 (6.7) |
| 2022 | Enteritidis 1 | Not resistant | None | 312/313 (99.7) |
|  |  |  | T | 1/313 (0.3) |
| 2022 | Enteritidis 2 | CRR only | Cip(I/R)Col | 1/90 (1.1) |
|  |  |  | Cip(I/R)Nal | 89/90 (98.9) |
| 2022 | Enteritidis 3 | CRR only | Cip(I/R)Nal | 113/113 (100) |
| 2022 | Enteritidis 4 | Not resistant | None | 43/43 (100) |
| 2022 | Hadar | Resistant | None | 4/267 (1.5) |
|  |  |  | Cip(I/R)Nal | 1/267 (0.4) |
|  |  |  | S | 5/267 (1.9) |
|  |  |  | SSuTGen | 1/267 (0.4) |
|  |  |  | ST | 253/267 (94.8) |
|  |  |  | STGen | 1/267 (0.4) |
|  |  |  | STKan | 1/267 (0.4) |
|  |  |  | T | 1/267 (0.4) |
| 2022 | Indiana | Not resistant | None | 21/21 (100) |
| 2022 | Infantis 1 | Not resistant | None | 52/57 (91.2) |
|  |  |  | ASSuTAuCxTioFoxGen | 1/57 (1.8) |
|  |  |  | SGen | 1/57 (1.8) |
|  |  |  | T | 3/57 (5.3) |
| 2022 | Infantis 2 | Not resistant | None | 100/108 (92.6) |
|  |  |  | AAuCxTioFox | 1/108 (0.9) |
|  |  |  | ASSuAuCxTioFoxGen | 1/108 (0.9) |
|  |  |  | CSSuT | 1/108 (0.9) |
|  |  |  | Kan | 1/108 (0.9) |
|  |  |  | SSuGen | 1/108 (0.9) |
|  |  |  | T | 3/108 (2.8) |
| 2022 | Mbandaka 1 | Not resistant | None | 25/26 (96.2) |
|  |  |  | T | 1/26 (3.8) |
| 2022 | Mbandaka 2 | Not resistant | None | 5/7 (71.4) |
|  |  |  | T | 2/7 (28.6) |
| 2022 | Typhimurium 1 | Not resistant | None | 39/43 (90.7) |
|  |  |  | ASuCotTmp | 4/43 (9.3) |
| 2022 | Typhimurium 2 | CRR only | None | 79/97 (81.4) |
|  |  |  | ASu | 1/97 (1.0) |
|  |  |  | ASuCotTmp | 14/97 (14.4) |
|  |  |  | SSuGen | 2/97 (2.1) |
|  |  |  | T | 1/97 (1.0) |
| 2022 | I 4,[5],12:i:- | Resistant | STGen | 1/12 (8.3) |
|  |  |  | T | 11/12 (91.7) |
| 2023 | Braenderup 1 | Resistant | None | 62/88 (70.5) |
|  |  |  | ASSuAuCxTioFoxGen | 1/88 (1.1) |
|  |  |  | SGen | 1/88 (1.1) |
|  |  |  | SSuGen | 1/88 (1.1) |
|  |  |  | STGen | 2/88 (2.3) |
|  |  |  | T | 21/88 (23.9) |
| 2023 | Braenderup 2 | CRR only | None | 59/69 (85.5) |
|  |  |  | A | 2/69 (2.9) |
|  |  |  | AAuCxTioFox | 6/69 (8.7) |
|  |  |  | STGen | 1/69 (1.4) |
|  |  |  | T | 1/69 (1.4) |
| 2023 | Enteritidis 1 | Not resistant | None | 30/30 (100) |
| 2023 | Enteritidis 2 | Not resistant | None | 109/111 (98.2) |
|  |  |  | Col | 1/111 (0.9) |
|  |  |  | Su | 1/111 (0.9) |
| 2023 | Enteritidis 3 | Not resistant | None | 210/212 (99.1) |
|  |  |  | Cip(I/R) | 1/212 (0.5) |
|  |  |  | Col | 1/212 (0.5) |
| 2023 | Enteritidis 4 | Not resistant | None | 24/24 (100) |
| 2023 | Indiana | Not resistant | None | 32/33 (97.0) |
|  |  |  | AAuCxTioFox | 1/33 (3.0) |
| 2023 | Infantis | Resistant | None | 98/121 (81.0) |
|  |  |  | AAuCxTioFox | 1/121 (0.8) |
|  |  |  | SGen | 4/121 (3.3) |
|  |  |  | SSuGen | 6/121 (5.0) |
|  |  |  | SSuTGen | 1/121 (0.8) |
|  |  |  | ST | 1/121 (0.8) |
|  |  |  | STGen | 1/121 (0.8) |
|  |  |  | T | 9/121 (7.4) |
| 2023 | Mbandaka 1 | Not resistant | None | 80/87 (92.0) |
|  |  |  | AAuCxTioFox | 1/87 (1.1) |
|  |  |  | Cip(I/R)Nal | 1/87 (1.1) |
|  |  |  | SGen | 3/87 (3.4) |
|  |  |  | SSuGen | 1/87 (1.1) |
|  |  |  | T | 1/87 (1.1) |
| 2023 | Mbandaka 2 | Not resistant | None | 59/61 (96.7) |
|  |  |  | T | 2/61 (3.3) |
| 2023 | Typhimurium 1 | Not resistant | None | 71/73 (97.3) |
|  |  |  | SGen | 1/73 (1.4) |
|  |  |  | T | 1/73 (1.4) |
| 2023 | Typhimurium 2 | Not resistant | None | 66/71 (93.0) |
|  |  |  | T | 5/71 (7.0) |
| 2023 | Typhimurium 3 | Not resistant | None | 6/6 (100) |

^a^CRR = Clinically relevant resistance; MDR = Multi-drug resistance

**^b^**A = ampicillin; Au = amoxicillin-clavulanic acid; Azm = azithromycin; C = chloramphenicol; Col = Colistin; Cip(I/R) = ciprofloxacin (intermediate or resistant); Cot = trimethoprim-sulfamethoxazole; Cx = ceftriaxone; Fos = fosfomycin; Fox = cefoxitin; Gen = gentamicin; Kan = kanamycin; Nal = nalidixic acid; S = streptomycin; Su = sulfisoxazole; T = tetracycline; Tio = Ceftiofur; Tmp = Trimethoprim

^c^Intrinsic resistance has been observed in some serotypes of *Salmonella* and is not associated with acquired resistance genes [e.g., *mcr-1*]; none of the BYPAS isolates possessed a colistin resistance gene.

**^d^**n/N (%)
