# Supplementary material for: Antimicrobial resistance in nontyphoidal Salmonella associated with multistate outbreaks linked to backyard poultry, United States, 2018–2023
Source: Front Public Health. 2026 Jun 12;14:1854943. doi: 10.3389/fpubh.2026.1854943 (PMC13303768; doi:10.3389/fpubh.2026.1854943)
Supplement: Supplementary file 3 [file Table_2.DOCX]

**Supplementary Table 2.** Resistance to antimicrobial classes and drugs by year in multistate BYP-associated nontyphoidal salmonellosis outbreaks – United States, 2018-2023.^a,b^

| **Antimicrobial Class** | **Antimicrobial Drug** | **2018–2023**  N = 2,248 | **2018**  N = 85 | **2019**  N = 215 | **2020**  N = 952 | **2021**  N = 399 | **2022**  N = 517 | **2023**  N = 80 |
| --- | --- | --- | --- | --- | --- | --- | --- | --- |
|  |  | **No. resistant / No. resistant isolates each year (%)** | | | | | | |
| Aminoglycosides | Streptomycin | 1,600 / 2,246 (71.2) | 24 / 85 (28.2) | 65 / 215 (30.2) | 843 / 952 (88.6) | 375 / 398 (94.2) | 269 / 516 (52.1) | 24 / 80 (30.0) |
|  | Gentamicin | 110 / 2,248 (4.9) | 7 / 85 (8.2) | 25 / 215 (11.6) | 24 / 952 (2.5) | 22 / 399 (5.5) | 9 / 517 (1.7) | 23 / 80 (28.8) |
|  | Kanamycin | 32 / 2,238 (1.4) | 0 / 85 (0) | 17 / 207 (8.2) | 8 / 952 (0.8) | 5 / 398 (1.3) | 2 / 516 (0.4) | 0 / 80 (0) |
|  | Total | 1,631 / 2,248 (72.6) | 24 / 85 (28.2) | 82 / 215 (38.1) | 850 / 952 (89.3) | 381 / 399 (95.5) | 270 / 517 (52.2) | 24 / 80 (30.0) |
| β–lactam combination agents | Amoxicillin-Clavulanic Acid | 101 / 2,248 (4.5) | 12 / 85 (14.1) | 44 / 215 (20.5) | 26 / 952 (2.7) | 6 / 399 (1.5) | 3 / 517 (0.6) | 10 / 80 (12.5) |
| Cephems | **Ceftriaxone** | **103 / 2,248 (4.6)** | **12 / 85 (14.1)** | **45 / 215 (20.9)** | **27 / 952 (2.8)** | **6 / 399 (1.5)** | **3 / 517 (0.6)** | **10 / 80 (12.5)** |
|  | Ceftiofur | 102 / 2,238 (4.6) | 12 / 85 (14.1) | 44 / 207 (21.3) | 27 / 952 (2.8) | 6 / 398 (1.5) | 3 / 516 (0.6) | 10 / 80 (12.5) |
|  | Cefoxitin | 101 / 2,248 (4.5) | 12 / 85 (14.1) | 44 / 215 (20.5) | 26 / 952 (2.7) | 6 / 399 (1.5) | 3 / 517 (0.6) | 10 / 80 (12.5) |
|  | Total | 103 / 2,248 (4.6) | 12 / 85 (14.1) | 45 / 215 (20.9) | 27 / 952 (2.8) | 6 / 399 (1.5) | 3 / 517 (0.6) | 10 / 80 (12.5) |
| Folate Pathway Antagonists | **Trimethoprim-Sulfamethoxazole** | **54 / 2,248 (2.4)** | **0 / 85 (0)** | **2 / 215 (0.9)** | **32 / 952 (3.4)** | **2 / 399 (0.5)** | **18 / 517 (3.5)** | **0 / 80 (0)** |
|  | Sulfisoxazole | 220 / 2,248 (9.8) | 19 / 85 (22.4) | 71 / 215 (33.0) | 69 / 952 (7.2) | 24 / 399 (6.0) | 26 / 517 (5.0) | 11 / 80 (13.8) |
|  | Trimethoprim | 55 / 2,244 (2.5) | 0 / 85 (0) | 2 / 207 (1.0) | 32 / 952 (3.4) | 3 / 398 (0.8) | 18 / 516 (3.5) | 0 / 80 (0) |
|  | Total | 221 / 2,248 (9.8) | 19 / 85 (22.4) | 71 / 215 (33.0) | 69 / 952 (7.2) | 25 / 399 (6.3) | 26 / 517 (5.0) | 11 / 80 (13.8) |
| Phosphonic Acids/Derivatives | Fosfomycin | 152 / 2,238 (6.8) | 54 / 85 (63.5) | 61 / 207 (29.5) | 37 / 952 (3.9) | 0 / 398 (0) | 0 / 516 (0) | 0 / 80 (0) |
| Penicillins | **Ampicillin** | **180 / 2,248 (8.0)** | **24 / 85 (28.2)** | **51 / 215 (23.7)** | **61 / 952 (6.4)** | **10 / 399 (2.5)** | **22 / 517 (4.3)** | **12 / 80 (15.0)** |
| Phenicols | Chloramphenicol | 57 / 2,248 (2.5) | 0 / 85 (0) | 40 / 215 (18.6) | 13 / 952 (1.4) | 3 / 399 (0.8) | 1 / 517 (0.2) | 0 / 80 (0) |
| Polymixins | Colistin^c^ | 4 / 2,240 (0.2) | 0 / 85 (0) | 0 / 207 (0) | 0 / 952 (0) | 1 / 399 (0.3) | 1 / 517 (0.2) | 2 / 80 (2.5) |
| Quinolones | **Ciprofloxacin**^d^ | **215 / 2,248 (9.6)** | **1 / 85 (1.2)** | **1 / 215 (0.5)** | **4 / 952 (0.4)** | **3 / 399 (0.8)** | **204 / 517 (39.5)** | **2 / 80 (2.5)** |
|  | Nalidixic Acid | 206 / 2,248 (9.2) | 1 / 85 (1.2) | 1 / 215 (0.5) | 0 / 952 (0) | 0 / 399 (0) | 203 / 517 (39.3) | 1 / 80 (1.3) |
|  | Total | 215 / 2,248 (9.6) | 1 / 85 (1.2) | 1 / 215 (0.5) | 4 / 952 (0.4) | 3 / 399 (0.8) | 204 / 517 (39.5) | 2 / 80 (2.5) |
| Tetracyclines | Tetracycline | 1,596 / 2,248 (71.0) | 19 / 85 (22.4) | 41 / 215 (19.1) | 845 / 952 (88.8) | 363 / 399 (91.0) | 282 / 517 (54.5) | 46 / 80 (57.5) |

^a^Amikacin, azithromycin, and meropenem were excluded from the table because none of the isolates were resistant to these antimicrobials. Bolded text reflects values for clinically relevant antimicrobials.

^b^Denominators for ceftiofur, colistin, fosfomycin, kanamycin, streptomycin, and trimethoprim were adjusted to exclude isolates for which no resistance results were available; these antimicrobials were not on the phenotypic testing panel for all years of the analysis.

^c^Intrinsic resistance has been observed in some serotypes of *Salmonella* and is not associated with acquired resistance genes [e.g., *mcr-1*]; none of the BYPAS isolates possessed colistin resistance genes.

^d^Includes isolates with ‘intermediate’ and ‘resistant’ ciprofloxacin resistance interpretations.
