# Supplementary material for: Antimicrobial resistance in nontyphoidal Salmonella associated with multistate outbreaks linked to backyard poultry, United States, 2018–2023
Source: Front Public Health. 2026 Jun 12;14:1854943. doi: 10.3389/fpubh.2026.1854943 (PMC13303768; doi:10.3389/fpubh.2026.1854943)
Supplement: Supplementary file 4 [file Table_3.DOCX]

**Supplementary Table 3.** Antimicrobial resistance in isolates from 78 multistate outbreaks of nontyphoidal *Salmonella* illnesses linked to backyard poultry contact, United States, 2018-2023 (n = 6,262 isolates).^a^

| Year | Serotype | Total Isolates | Amoxicillin-Clavulanic Acid | **Ampicillin** | Cefoxitin | Ceftiofur | **Ceftriaxone** | Chloramphenicol | **Ciprofloxacin**^b^ | Colistin^c^ | Fosfomycin | Gentamicin | Kanamycin | Nalidixic Acid | Streptomycin | Sulfisoxazole | Tetracycline | Trimethoprim | **Trimethoprim-Sulfamethoxazole** |
| --- | --- | --- | --- | --- | --- | --- | --- | --- | --- | --- | --- | --- | --- | --- | --- | --- | --- | --- | --- |
|  |  |  | No. (%) | | | | | | | | | | | | | | | | |
| 2018 | Enteritidis 1 | 52 | 0 (0) | **0 (0)** | 0 (0) | 0 (0) | **0 (0)** | 0 (0) | **0 (0)** | 0 (0) | 0 (0) | 0 (0) | 0 (0) | 0 (0) | 0 (0) | 0 (0) | 0 (0) | 0 (0) | **0 (0)** |
| 2018 | Enteritidis 2 | 66 | 0 (0) | **12 (18.2)** | 0 (0) | 0 (0) | **0 (0)** | 0 (0) | **1 (1.5)** | 0 (0) | 0 (0) | 0 (0) | 0 (0) | 1 (1.5) | 11 (16.7) | 11 (16.7) | 11 (16.7) | 0 (0) | **0 (0)** |
| 2018 | Indiana | 15 | 0 (0) | **0 (0)** | 0 (0) | 0 (0) | **0 (0)** | 0 (0) | **0 (0)** | 0 (0) | 0 (0) | 0 (0) | 0 (0) | 0 (0) | 0 (0) | 0 (0) | 0 (0) | 0 (0) | **0 (0)** |
| 2018 | Infantis | 67 | 8 (11.9) | **8 (11.9)** | 8 (11.9) | 8 (11.9) | **8 (11.9)** | 0 (0) | **0 (0)** | 0 (0) | 0 (0) | 2 (3) | 0 (0) | 0 (0) | 4 (6) | 3 (4.5) | 0 (0) | 0 (0) | **0 (0)** |
| 2018 | Litchfield | 9 | 1 (11.1) | **1 (11.1)** | 1 (11.1) | 1 (11.1) | **1 (11.1)** | 0 (0) | **0 (0)** | 0 (0) | 0 (0) | 1 (11.1) | 0 (0) | 0 (0) | 5 (55.6) | 1 (11.1) | 6 (66.7) | 0 (0) | **0 (0)** |
| 2018 | Montevideo 1 | 38 | 3 (7.9) | **3 (7.9)** | 3 (7.9) | 3 (7.9) | **3 (7.9)** | 0 (0) | **0 (0)** | 0 (0) | 37 (97.4) | 2 (5.3) | 0 (0) | 0 (0) | 2 (5.3) | 2 (5.3) | 2 (5.3) | 0 (0) | **0 (0)** |
| 2018 | Montevideo 2 | 19 | 0 (0) | **0 (0)** | 0 (0) | 0 (0) | **0 (0)** | 0 (0) | **0 (0)** | 0 (0) | 17 (89.5) | 2 (10.5) | 0 (0) | 0 (0) | 2 (10.5) | 2 (10.5) | 0 (0) | 0 (0) | **0 (0)** |
| 2018 | Senftenberg | 28 | 0 (0) | **0 (0)** | 0 (0) | 0 (0) | **0 (0)** | 0 (0) | **0 (0)** | 0 (0) | 0 (0) | 0 (0) | 0 (0) | 0 (0) | 0 (0) | 0 (0) | 0 (0) | 0 (0) | **0 (0)** |
| 2019 | Agona | 50 | 0 (0) | **0 (0)** | 0 (0) | 0 (0) | **0 (0)** | 0 (0) | **0 (0)** | 0 (0) | 44 (88.0) | 0 (0) | 0 (0) | 0 (0) | 0 (0) | 0 (0) | 0 (0) | 0 (0) | **0 (0)** |
| 2019 | Alachua | 13 | 1 (7.7) | **3 (23.1)** | 1 (7.7) | 0 (0) | **1 (7.7)** | 0 (0) | **0 (0)** | 0 (0) | 10 (76.9) | 1 (7.7) | 0 (0) | 0 (0) | 1 (7.7) | 1 (7.7) | 1 (7.7) | 0 (0) | **0 (0)** |
| 2019 | Altona | 7 | 0 (0) | **0 (0)** | 0 (0) | 0 (0) | **0 (0)** | 1 (14.3) | **0 (0)** | 0 (0) | 0 (0) | 0 (0) | 2 (28.6) | 0 (0) | 1 (14.3) | 1 (14.3) | 3 (42.9) | 0 (0) | **0 (0)** |
| 2019 | Anatum | 68 | 9 (13.2) | **9 (13.2)** | 9 (13.2) | 9 (13.2) | **9 (13.2)** | 1 (1.5) | **1 (1.5)** | 0 (0) | 0 (0) | 0 (0) | 0 (0) | 1 (1.5) | 1 (1.5) | 1 (1.5) | 2 (2.9) | 0 (0) | **0 (0)** |
| 2019 | Braenderup 1 | 147 | 15 (10.2) | **17 (11.6)** | 15 (10.2) | 15 (10.2) | **15 (10.2)** | 0 (0) | **0 (0)** | 0 (0) | 0 (0) | 2 (1.4) | 0 (0) | 0 (0) | 3 (2.0) | 4 (2.7) | 9 (6.1) | 2 (1.4) | **2 (1.4)** |
| 2019 | Braenderup 2 | 12 | 0 (0) | **0 (0)** | 0 (0) | 0 (0) | **0 (0)** | 0 (0) | **0 (0)** | 0 (0) | 0 (0) | 2 (16.7) | 0 (0) | 0 (0) | 2 (16.7) | 2 (16.7) | 3 (25.0) | 0 (0) | **0 (0)** |
| 2019 | Enteritidis 1 | 255 | 0 (0) | **0 (0)** | 0 (0) | 0 (0) | **0 (0)** | 0 (0) | **0 (0)** | 0 (0) | 0 (0) | 0 (0) | 0 (0) | 0 (0) | 0 (0) | 0 (0) | 1 (0.4) | 0 (0) | **0 (0)** |
| 2019 | Enteritidis 2 | 139 | 0 (0) | **0 (0)** | 0 (0) | 0 (0) | **0 (0)** | 0 (0) | **0 (0)** | 0 (0) | 0 (0) | 0 (0) | 0 (0) | 0 (0) | 0 (0) | 0 (0) | 0 (0) | 0 (0) | **0 (0)** |
| 2019 | Infantis 1 | 119 | 14 (11.8) | **16 (13.4)** | 14 (11.8) | 14 (11.8) | **14 (11.8)** | 3 (2.5) | **0 (0)** | 0 (0) | 0 (0) | 15 (12.6) | 9 (7.6) | 0 (0) | 18 (15.1) | 21 (17.6) | 13 (10.9) | 0 (0) | **0 (0)** |
| 2019 | Infantis 2 | 19 | 0 (0) | **0 (0)** | 0 (0) | 0 (0) | **0 (0)** | 0 (0) | **0 (0)** | 0 (0) | 0 (0) | 3 (15.8) | 6 (31.6) | 0 (0) | 3 (15.8) | 3 (15.8) | 3 (15.8) | 0 (0) | **0 (0)** |
| 2019 | Manhattan | 36 | 0 (0) | **0 (0)** | 0 (0) | 0 (0) | **0 (0)** | 34 (94.4) | **0 (0)** | 0 (0) | 0 (0) | 0 (0) | 0 (0) | 0 (0) | 33 (91.7) | 34 (94.4) | 0 (0) | 0 (0) | **0 (0)** |
| 2019 | Montevideo | 9 | 0 (0) | **0 (0)** | 0 (0) | 0 (0) | **0 (0)** | 0 (0) | **0 (0)** | 0 (0) | 7 (77.8) | 0 (0) | 0 (0) | 0 (0) | 0 (0) | 0 (0) | 0 (0) | 0 (0) | **0 (0)** |
| 2019 | Muenchen | 15 | 0 (0) | **0 (0)** | 0 (0) | 0 (0) | **0 (0)** | 0 (0) | **0 (0)** | 0 (0) | 0 (0) | 0 (0) | 0 (0) | 0 (0) | 0 (0) | 0 (0) | 0 (0) | 0 (0) | **0 (0)** |
| 2019 | Newport | 68 | 5 (7.4) | **6 (8.8)** | 5 (7.4) | 6 (8.8) | **6 (8.8)** | 1 (1.5) | **0 (0)** | 0 (0) | 0 (0) | 2 (2.9) | 0 (0) | 0 (0) | 3 (4.4) | 4 (5.9) | 6 (8.8) | 0 (0) | **0 (0)** |
| 2019 | Oranienburg | 9 | 0 (0) | **0 (0)** | 0 (0) | 0 (0) | **0 (0)** | 0 (0) | **0 (0)** | 0 (0) | 0 (0) | 0 (0) | 0 (0) | 0 (0) | 0 (0) | 0 (0) | 0 (0) | 0 (0) | **0 (0)** |
| 2020 | Agona | 37 | 0 (0) | **0 (0)** | 0 (0) | 0 (0) | **0 (0)** | 0 (0) | **0 (0)** | 0 (0) | 37 (100) | 0 (0) | 0 (0) | 0 (0) | 0 (0) | 0 (0) | 0 (0) | 0 (0) | **0 (0)** |
| 2020 | Anatum | 40 | 10 (25.0) | **10 (25.0)** | 10 (25.0) | 10 (25.0) | **10 (25.0)** | 0 (0) | **0 (0)** | 0 (0) | 0 (0) | 0 (0) | 1 (2.5) | 0 (0) | 0 (0) | 0 (0) | 0 (0) | 0 (0) | **0 (0)** |
| 2020 | Braenderup | 54 | 0 (0) | **1 (1.9)** | 0 (0) | 1 (1.9) | **1 (1.9)** | 1 (1.9) | **0 (0)** | 0 (0) | 0 (0) | 0 (0) | 3 (5.6) | 0 (0) | 1 (1.9) | 2 (3.7) | 10 (18.5) | 0 (0) | **0 (0)** |
| 2020 | Enteritidis 1 | 132 | 0 (0) | **0 (0)** | 0 (0) | 0 (0) | **0 (0)** | 0 (0) | **1 (0.8)** | 0 (0) | 0 (0) | 0 (0) | 0 (0) | 0 (0) | 0 (0) | 0 (0) | 0 (0) | 0 (0) | **0 (0)** |
| 2020 | Enteritidis 2 | 34 | 0 (0) | **0 (0)** | 0 (0) | 0 (0) | **0 (0)** | 0 (0) | **0 (0)** | 0 (0) | 0 (0) | 0 (0) | 0 (0) | 0 (0) | 0 (0) | 0 (0) | 0 (0) | 0 (0) | **0 (0)** |
| 2020 | Enteritidis 3 | 155 | 0 (0) | **0 (0)** | 0 (0) | 0 (0) | **0 (0)** | 0 (0) | **0 (0)** | 0 (0) | 0 (0) | 0 (0) | 0 (0) | 0 (0) | 0 (0) | 0 (0) | 0 (0) | 0 (0) | **0 (0)** |
| 2020 | Enteritidis 4 | 66 | 0 (0) | **1 (1.5)** | 0 (0) | 0 (0) | **0 (0)** | 1 (1.5) | **0 (0)** | 0 (0) | 0 (0) | 0 (0) | 0 (0) | 0 (0) | 0 (0) | 0 (0) | 1 (1.5) | 0 (0) | **0 (0)** |
| 2020 | Hadar | 835 | 7 (0.8) | **9 (1.1)** | 7 (0.8) | 7 (0.8) | **7 (0.8)** | 9 (1.1) | **2 (0.2)** | 0 (0) | 0 (0) | 14 (1.7) | 1 (0.1) | 0 (0) | 827 (99.0) | 24 (2.9) | 820 (98.2) | 2 (0.2) | **2 (0.2)** |
| 2020 | Infantis 1 | 80 | 2 (2.5) | **2 (2.5)** | 2 (2.5) | 2 (2.5) | **2 (2.5)** | 0 (0) | **0 (0)** | 0 (0) | 0 (0) | 2 (2.5) | 1 (1.2) | 0 (0) | 2 (2.5) | 3 (3.8) | 3 (3.8) | 0 (0) | **0 (0)** |
| 2020 | Infantis 2 | 32 | 3 (9.4) | **3 (9.4)** | 3 (9.4) | 3 (9.4) | **3 (9.4)** | 0 (0) | **0 (0)** | 0 (0) | 0 (0) | 1 (3.1) | 1 (3.1) | 0 (0) | 4 (12.5) | 1 (3.1) | 2 (6.2) | 0 (0) | **0 (0)** |
| 2020 | Mbandaka | 38 | 3 (7.9) | **3 (7.9)** | 3 (7.9) | 3 (7.9) | **3 (7.9)** | 0 (0) | **0 (0)** | 0 (0) | 0 (0) | 0 (0) | 1 (2.6) | 0 (0) | 0 (0) | 0 (0) | 2 (5.3) | 0 (0) | **0 (0)** |
| 2020 | Muenchen | 26 | 0 (0) | **0 (0)** | 0 (0) | 0 (0) | **0 (0)** | 0 (0) | **0 (0)** | 0 (0) | 0 (0) | 0 (0) | 0 (0) | 0 (0) | 0 (0) | 0 (0) | 0 (0) | 0 (0) | **0 (0)** |
| 2020 | Newport | 28 | 1 (3.6) | **1 (3.6)** | 1 (3.6) | 1 (3.6) | **1 (3.6)** | 0 (0) | **0 (0)** | 0 (0) | 0 (0) | 2 (7.1) | 0 (0) | 0 (0) | 2 (7.1) | 2 (7.1) | 1 (3.6) | 0 (0) | **0 (0)** |
| 2020 | Thompson | 22 | 0 (0) | **0 (0)** | 0 (0) | 0 (0) | **0 (0)** | 0 (0) | **0 (0)** | 0 (0) | 0 (0) | 1 (4.5) | 0 (0) | 0 (0) | 1 (4.5) | 1 (4.5) | 2 (9.1) | 0 (0) | **0 (0)** |
| 2020 | Typhimurium 1 | 28 | 0 (0) | **23 (82.1)** | 0 (0) | 0 (0) | **0 (0)** | 0 (0) | **1 (3.6)** | 0 (0) | 0 (0) | 0 (0) | 0 (0) | 0 (0) | 0 (0) | 23 (82.1) | 0 (0) | 23 (82.1) | **23 (82.1)** |
| 2020 | Typhimurium 2 | 43 | 0 (0) | **3 (7.0)** | 0 (0) | 0 (0) | **0 (0)** | 0 (0) | **0 (0)** | 0 (0) | 0 (0) | 3 (7.0) | 0 (0) | 0 (0) | 3 (7.0) | 6 (14.0) | 2 (4.7) | 3 (7.0) | **3 (7.0)** |
| 2020 | I 4,[5],12:i:- | 54 | 0 (0) | **5 (9.3)** | 0 (0) | 0 (0) | **0 (0)** | 2 (3.7) | **0 (0)** | 0 (0) | 0 (0) | 1 (1.9) | 0 (0) | 0 (0) | 3 (5.6) | 7 (13.0) | 2 (3.7) | 4 (7.4) | **4 (7.4)** |
| 2021 | Enteritidis 1 | 130 | 0 (0) | **0 (0)** | 0 (0) | 0 (0) | **0 (0)** | 0 (0) | **0 (0)** | 0 (0) | 0 (0) | 1 (0.8) | 0 (0) | 0 (0) | 1 (0.8) | 0 (0) | 1 (0.8) | 0 (0) | **0 (0)** |
| 2021 | Enteritidis 2 | 92 | 0 (0) | **0 (0)** | 0 (0) | 0 (0) | **0 (0)** | 0 (0) | **0 (0)** | 0 (0) | 0 (0) | 0 (0) | 0 (0) | 0 (0) | 0 (0) | 0 (0) | 1 (1.1) | 0 (0) | **0 (0)** |
| 2021 | Enteritidis 3 | 21 | 0 (0) | **0 (0)** | 0 (0) | 0 (0) | **0 (0)** | 0 (0) | **0 (0)** | 0 (0) | 0 (0) | 0 (0) | 0 (0) | 0 (0) | 0 (0) | 0 (0) | 0 (0) | 0 (0) | **0 (0)** |
| 2021 | Enteritidis 4 | 49 | 0 (0) | **0 (0)** | 0 (0) | 0 (0) | **0 (0)** | 0 (0) | **0 (0)** | 0 (0) | 0 (0) | 0 (0) | 0 (0) | 0 (0) | 0 (0) | 0 (0) | 0 (0) | 0 (0) | **0 (0)** |
| 2021 | Enteritidis 5 | 27 | 0 (0) | **0 (0)** | 0 (0) | 0 (0) | **0 (0)** | 0 (0) | **0 (0)** | 1 (3.7) | 0 (0) | 0 (0) | 0 (0) | 0 (0) | 0 (0) | 0 (0) | 0 (0) | 0 (0) | **0 (0)** |
| 2021 | Enteritidis 6 | 39 | 0 (0) | **0 (0)** | 0 (0) | 0 (0) | **0 (0)** | 0 (0) | **1 (2.6)** | 0 (0) | 0 (0) | 0 (0) | 0 (0) | 0 (0) | 0 (0) | 0 (0) | 0 (0) | 1 (2.6) | **0 (0)** |
| 2021 | Hadar | 359 | 0 (0) | **2 (0.6)** | 0 (0) | 0 (0) | **0 (0)** | 0 (0) | **0 (0)** | 0 (0) | 0 (0) | 1 (0.3) | 0 (0) | 0 (0) | 351 (97.8) | 3 (0.8) | 349 (97.2) | 2 (0.6) | **2 (0.6)** |
| 2021 | Indiana | 8 | 0 (0) | **0 (0)** | 0 (0) | 0 (0) | **0 (0)** | 0 (0) | **0 (0)** | 0 (0) | 0 (0) | 0 (0) | 0 (0) | 0 (0) | 0 (0) | 0 (0) | 0 (0) | 0 (0) | **0 (0)** |
| 2021 | Infantis 1 | 283 | 5 (1.8) | **7 (2.5)** | 5 (1.8) | 5 (1.8) | **5 (1.8)** | 3 (1.1) | **2 (0.7)** | 0 (0) | 0 (0) | 8 (2.8) | 3 (1.1) | 0 (0) | 11 (3.9) | 11 (3.9) | 7 (2.5) | 0 (0) | **0 (0)** |
| 2021 | Infantis 2 | 52 | 1 (1.9) | **1 (1.9)** | 1 (1.9) | 1 (1.9) | **1 (1.9)** | 0 (0) | **0 (0)** | 0 (0) | 0 (0) | 11 (21.2) | 2 (3.8) | 0 (0) | 11 (21.2) | 10 (19.2) | 3 (5.8) | 0 (0) | **0 (0)** |
| 2021 | Mbandaka | 40 | 0 (0) | **0 (0)** | 0 (0) | 0 (0) | **0 (0)** | 0 (0) | **0 (0)** | 0 (0) | 0 (0) | 1 (2.5) | 0 (0) | 0 (0) | 1 (2.5) | 0 (0) | 1 (2.5) | 0 (0) | **0 (0)** |
| 2021 | Muenchen | 15 | 0 (0) | **0 (0)** | 0 (0) | 0 (0) | **0 (0)** | 0 (0) | **0 (0)** | 0 (0) | 0 (0) | 0 (0) | 0 (0) | 0 (0) | 0 (0) | 0 (0) | 1 (6.7) | 0 (0) | **0 (0)** |
| 2022 | Enteritidis 1 | 313 | 0 (0) | **0 (0)** | 0 (0) | 0 (0) | **0 (0)** | 0 (0) | **0 (0)** | 0 (0) | 0 (0) | 0 (0) | 0 (0) | 0 (0) | 0 (0) | 0 (0) | 1 (0.3) | 0 (0) | **0 (0)** |
| 2022 | Enteritidis 2 | 90 | 0 (0) | **0 (0)** | 0 (0) | 0 (0) | **0 (0)** | 0 (0) | **90 (100)** | 1 (1.1) | 0 (0) | 0 (0) | 0 (0) | 89 (98.9) | 0 (0) | 0 (0) | 0 (0) | 0 (0) | **0 (0)** |
| 2022 | Enteritidis 3 | 113 | 0 (0) | **0 (0)** | 0 (0) | 0 (0) | **0 (0)** | 0 (0) | **113 (100)** | 0 (0) | 0 (0) | 0 (0) | 0 (0) | 113 (100) | 0 (0) | 0 (0) | 0 (0) | 0 (0) | **0 (0)** |
| 2022 | Enteritidis 4 | 43 | 0 (0) | **0 (0)** | 0 (0) | 0 (0) | **0 (0)** | 0 (0) | **0 (0)** | 0 (0) | 0 (0) | 0 (0) | 0 (0) | 0 (0) | 0 (0) | 0 (0) | 0 (0) | 0 (0) | **0 (0)** |
| 2022 | Hadar | 267 | 0 (0) | **0 (0)** | 0 (0) | 0 (0) | **0 (0)** | 0 (0) | **1 (0.4)** | 0 (0) | 0 (0) | 2 (0.7) | 1 (0.4) | 1 (0.4) | 261 (97.8) | 1 (0.4) | 257 (96.3) | 0 (0) | **0 (0)** |
| 2022 | Indiana | 21 | 0 (0) | **0 (0)** | 0 (0) | 0 (0) | **0 (0)** | 0 (0) | **0 (0)** | 0 (0) | 0 (0) | 0 (0) | 0 (0) | 0 (0) | 0 (0) | 0 (0) | 0 (0) | 0 (0) | **0 (0)** |
| 2022 | Infantis 1 | 57 | 1 (1.8) | **1 (1.8)** | 1 (1.8) | 1 (1.8) | **1 (1.8)** | 0 (0) | **0 (0)** | 0 (0) | 0 (0) | 2 (3.5) | 0 (0) | 0 (0) | 2 (3.5) | 1 (1.8) | 4 (7.0) | 0 (0) | **0 (0)** |
| 2022 | Infantis 2 | 108 | 2 (1.9) | **2 (1.9)** | 2 (1.9) | 2 (1.9) | **2 (1.9)** | 1 (0.9) | **0 (0)** | 0 (0) | 0 (0) | 2 (1.9) | 1 (0.9) | 0 (0) | 3 (2.8) | 3 (2.8) | 4 (3.7) | 0 (0) | **0 (0)** |
| 2022 | Mbandaka 1 | 26 | 0 (0) | **0 (0)** | 0 (0) | 0 (0) | **0 (0)** | 0 (0) | **0 (0)** | 0 (0) | 0 (0) | 0 (0) | 0 (0) | 0 (0) | 0 (0) | 0 (0) | 1 (3.8) | 0 (0) | **0 (0)** |
| 2022 | Mbandaka 2 | 7 | 0 (0) | **0 (0)** | 0 (0) | 0 (0) | **0 (0)** | 0 (0) | **0 (0)** | 0 (0) | 0 (0) | 0 (0) | 0 (0) | 0 (0) | 0 (0) | 0 (0) | 2 (28.6) | 0 (0) | **0 (0)** |
| 2022 | Typhimurium 1 | 43 | 0 (0) | **4 (9.3)** | 0 (0) | 0 (0) | **0 (0)** | 0 (0) | **0 (0)** | 0 (0) | 0 (0) | 0 (0) | 0 (0) | 0 (0) | 0 (0) | 4 (9.3) | 0 (0) | 4 (9.3) | **4 (9.3)** |
| 2022 | Typhimurium 2 | 97 | 0 (0) | **15 (15.5)** | 0 (0) | 0 (0) | **0 (0)** | 0 (0) | **0 (0)** | 0 (0) | 0 (0) | 2 (2.1) | 0 (0) | 0 (0) | 2 (2.1) | 17 (17.5) | 1 (1.0) | 14 (14.4) | **14 (14.4)** |
| 2022 | I 4,[5],12:i:- | 12 | 0 (0) | **0 (0)** | 0 (0) | 0 (0) | **0 (0)** | 0 (0) | **0 (0)** | 0 (0) | 0 (0) | 1 (8.3) | 0 (0) | 0 (0) | 1 (8.3) | 0 (0) | 12 (100) | 0 (0) | **0 (0)** |
| 2023 | Braenderup 1 | 88 | 1 (1.1) | **1 (1.1)** | 1 (1.1) | 1 (1.1) | **1 (1.1)** | 0 (0) | **0 (0)** | 0 (0) | 0 (0) | 5 (5.7) | 0 (0) | 0 (0) | 5 (5.7) | 2 (2.3) | 23 (26.1) | 0 (0) | **0 (0)** |
| 2023 | Braenderup 2 | 69 | 6 (8.7) | **8 (11.6)** | 6 (8.7) | 6 (8.7) | **6 (8.7)** | 0 (0) | **0 (0)** | 0 (0) | 0 (0) | 1 (1.4) | 0 (0) | 0 (0) | 1 (1.4) | 0 (0) | 2 (2.9) | 0 (0) | **0 (0)** |
| 2023 | Enteritidis 1 | 30 | 0 (0) | **0 (0)** | 0 (0) | 0 (0) | **0 (0)** | 0 (0) | **0 (0)** | 0 (0) | 0 (0) | 0 (0) | 0 (0) | 0 (0) | 0 (0) | 0 (0) | 0 (0) | 0 (0) | **0 (0)** |
| 2023 | Enteritidis 2 | 111 | 0 (0) | **0 (0)** | 0 (0) | 0 (0) | **0 (0)** | 0 (0) | **0 (0)** | 1 (0.9) | 0 (0) | 0 (0) | 0 (0) | 0 (0) | 0 (0) | 1 (0.9) | 0 (0) | 0 (0) | **0 (0)** |
| 2023 | Enteritidis 3 | 212 | 0 (0) | **0 (0)** | 0 (0) | 0 (0) | **0 (0)** | 0 (0) | **1 (0.5)** | 1 (0.5) | 0 (0) | 0 (0) | 0 (0) | 0 (0) | 0 (0) | 0 (0) | 0 (0) | 0 (0) | **0 (0)** |
| 2023 | Enteritidis 4 | 24 | 0 (0) | **0 (0)** | 0 (0) | 0 (0) | **0 (0)** | 0 (0) | **0 (0)** | 0 (0) | 0 (0) | 0 (0) | 0 (0) | 0 (0) | 0 (0) | 0 (0) | 0 (0) | 0 (0) | **0 (0)** |
| 2023 | Indiana | 33 | 1 (3.0) | **1 (3.0)** | 1 (3.0) | 1 (3.0) | **1 (3.0)** | 0 (0) | **0 (0)** | 0 (0) | 0 (0) | 0 (0) | 0 (0) | 0 (0) | 0 (0) | 0 (0) | 0 (0) | 0 (0) | **0 (0)** |
| 2023 | Infantis | 121 | 1 (0.8) | **1 (0.8)** | 1 (0.8) | 1 (0.8) | **1 (0.8)** | 0 (0) | **0 (0)** | 0 (0) | 0 (0) | 12 (9.9) | 0 (0) | 0 (0) | 13 (10.7) | 7 (5.8) | 12 (9.9) | 0 (0) | **0 (0)** |
| 2023 | Mbandaka 1 | 87 | 1 (1.1) | **1 (1.1)** | 1 (1.1) | 1 (1.1) | **1 (1.1)** | 0 (0) | **1 (1.1)** | 0 (0) | 0 (0) | 4 (4.6) | 0 (0) | 1 (1.1) | 4 (4.6) | 1 (1.1) | 1 (1.1) | 0 (0) | **0 (0)** |
| 2023 | Mbandaka 2 | 61 | 0 (0) | **0 (0)** | 0 (0) | 0 (0) | **0 (0)** | 0 (0) | **0 (0)** | 0 (0) | 0 (0) | 0 (0) | 0 (0) | 0 (0) | 0 (0) | 0 (0) | 2 (3.3) | 0 (0) | **0 (0)** |
| 2023 | Typhimurium 1 | 73 | 0 (0) | **0 (0)** | 0 (0) | 0 (0) | **0 (0)** | 0 (0) | **0 (0)** | 0 (0) | 0 (0) | 1 (1.4) | 0 (0) | 0 (0) | 1 (1.4) | 0 (0) | 1 (1.4) | 0 (0) | **0 (0)** |
| 2023 | Typhimurium 2 | 71 | 0 (0) | **0 (0)** | 0 (0) | 0 (0) | **0 (0)** | 0 (0) | **0 (0)** | 0 (0) | 0 (0) | 0 (0) | 0 (0) | 0 (0) | 0 (0) | 0 (0) | 5 (7.0) | 0 (0) | **0 (0)** |
| 2023 | Typhimurium 3 | 6 | 0 (0) | **0 (0)** | 0 (0) | 0 (0) | **0 (0)** | 0 (0) | **0 (0)** | 0 (0) | 0 (0) | 0 (0) | 0 (0) | 0 (0) | 0 (0) | 0 (0) | 0 (0) | 0 (0) | **0 (0)** |
| Total  No. (%) | | 6,262 (100) | 101 (1.6) | **180 (2.9)** | 101 (1.6) | 102 (1.6) | **103 (1.6)** | 57 (0.9) | **215 (3.4)** | 4 (0.1) | 152 (2.4) | 110 (1.8) | 32 (0.5) | 206 (3.3) | 1600 (25.6) | 220 (3.5) | 1596 (25.5) | 55 (0.9) | **180 (2.9)** |

^a^Bolded text reflects values of clinically relevant antimicrobials. Amikacin, azithromycin, and meropenem were excluded from the table because none of the isolates were resistant to these antimicrobials

^b^Includes isolates with ‘intermediate’ and ‘resistant’ ciprofloxacin resistance interpretations.

^c^Intrinsic resistance has been observed in some serotypes of *Salmonella* and is not associated with acquired resistance genes [e.g., *mcr-1*]; none of the BYPAS isolates possessed *colistin resistance genes.*
